# The Inhibition Effect of Linezolid With Reyanning Mixture on MRSA and its Biofilm is More Significant than That of Linezolid Alone

**DOI:** 10.3389/fphar.2021.766309

**Published:** 2022-01-03

**Authors:** Lulu Zhang, Weifeng Yang, Yajun Chu, Bo Wen, Yungchi Cheng, Tariq Mahmood, Mei Bao, Feng Ge, Li Li, Jianfeng Yi, Chengqiang Du, Cheng Lu, Yong Tan

**Affiliations:** ^1^ Institute of Basic Research in Clinical Medicine, China Academy of Chinese Medical Sciences, Beijing, China; ^2^ Key Laboratory for Research on Active Ingredients in Natural Medicine of Jiangxi Province, Yichun University, Yichun, China; ^3^ Experimental Research Center, China Academy of Chinese Medical Sciences, Beijing, China; ^4^ Tsing Hua De Ren Xi an Happiness Pharmaceutical Co., Ltd., Xi’an, China; ^5^ Department of Pharmacology, Yale University School of Medicine, New Haven, CT, United States; ^6^ Department of Plant Sciences, Faculty of Biological Sciences, Quaid-i-Azam University, Islamabad, Pakistan; ^7^ Faculty of Life Science and Technology, Kunming University of Science and Technology, Kunming, China

**Keywords:** methicillin-resistant *Staphylococcus aureus*, biofilm, reyanning mixture, linezolid, synergistic inhibitory effect

## Abstract

Methicillin-resistant *Staphylococcus aureus* (MRSA) is a superbacterium, and when it forms biofilms, it is difficult to treat even with the first-line of antibiotic linezolid (LNZ). Reyanning mixture (RYN), a compound-based Chinese medicine formula, has been found to have inhibitory effects on biofilms. This study aims to explore the synergistic inhibitory effect and corresponding mechanisms of their (LNZ&RYN) combination on the planktonic as well as biofilm cells of MRSA. Broth microdilution and chessboard methods were employed for the determination of minimum inhibitory concentrations (MICs) and synergistic concentration of LNZ&RYN, respectively. The effect of the combined medication on biofilm and mature biofilm of MRSA were observed by biofilm morphology and permeability experiments, respectively. To unveil the molecular mechanism of action of the synergistic combination of LNZ and RYN, RT-PCR based biofilm-related gene expression analysis and ultra-high pressure liquid chromatography-time-of-flight mass spectrometry based endogenous metabonomic analysis were deployed. The results indicated that 1/16RYN as the best combined dose reduced LNZ (4 μg/ml) to 2 μg/ml. The combined treatment inhibited living MRSA before and after biofilm formation, removed the residual structure of dead bacteria in MRSA biofilms and affected the shape and size of bacteria, resulting in the improvement of biofilm permeability. The mechanism was that biofilm-related genes such as *agrC*, *atlA*, and *sarA*, as well as amino acid uptake associated with the metabolism of 3-dehydrocarnitine, kynurenine, *L*-leucine, *L*-lysine and sebacic acid were inhibited. This study provides evidence for the treatment of MRSA and its biofilms with LNZ combined with RYN.

## Introduction

Methicillin-resistant *Staphylococcus aureus* (MRSA) is commonly known as a “superbug”, and its infection leads to many refractory diseases such as pneumonia, bacteraemia, and skin and soft tissue infections (Patel, 2009). A clinical survey has shown that MRSA is highly prevalent in hospitals around the world, and the incidence of MRSA is more than 50% in Asia, North America and South America (Stefani et al., 2012). MRSA is difficult to treat, and when it forms a mature biofilm, its treatment will be more difficult. Biofilms are a structural group attached to living or inert surfaces formed by microbial cells that adhere to each other and are surrounded by their own extracellular polymeric substances (EPSs) to form a special membrane structure (Wu et al., 2015). As a natural barrier, the formation of biofilms is considered a way for microorganisms to adapt to harsh environments. Compared with planktonic bacteria, the resistance of mature biofilm bacteria to antibiotics and host immune defence is significantly improved and even antibiotics up to 100–1000 times the minimum inhibitory concentration (MIC) cannot kill bacteria in biofilms (David, 2003). Biofilms are one of the important reasons for MRSA resistance. More than 80% of bacterial infections are biofilm-mediated (Stella et al., 2021). How to destroy the structure of biofilms and effectively treat the related infections caused by MRSA and its biofilms has become a hotspot of scientific research.

Antibiotics are the primary and conventional drugs to treat MRSA infection. vancomycin is the first choice for the treatment of MRSA infection. With the long-term clinical use of Vancomycin, *S. aureus* with low sensitivity or even drug resistance to vancomycin has appeared (Gowrishankar et al., 2013). Linezolid (LNZ) is generally considered an alternative to vancomycin. It is an oxazolidinone antibiotic that inhibits protein synthesis by binding to 50 s ribosomal subunits (Wunderink et al., 2012). LNZ may be more effective in the treatment of hospital-acquired MRSA and ventilator-associated pneumonia than vancomycin (Wunderink et al., 2012). However, the treatment window of LNZ is narrow, and the probability of nephrotoxic adverse reactions is high. Moreover, MRSA has gradually become resistant to LNZ (Boudet et al., 2021).

Antibiotic resistance is currently a major challenge in antimicrobial therapy. There is a worldwide search for antibacterial products that can replace or compensate for the lack of antibiotics (Gowrishankar et al., 2015). Traditional plant medicine (TPM) has been used in the clinic for thousands of years. Many TPM extracts can regulate the quorum sensing (QS) system *in vitro* and reduce the adhesion ability of bacteria to inhibit the growth of bacteria and their biofilms (Karbasizade et al., 2017). However, the bacteriostatic ability of TPM is weaker than that of antibiotics. Through the complementary advantages of TPM and specific antibiotics, together they are expected to achieve better therapeutic effects. Studies have shown that when TPM is used in combination with antibiotics, TPM can inhibit and destroy drug-resistant biofilms; as a result, bacteria in biofilms can effectively contact antibiotics, and thus, the antibiotics kill bacteria to a greater extent (Dey et al., 2020). The Reyanning mixture (ReYanNingHeJi, RYN), a Chinese patent drug, is composed of Taraxacum, Polygonum cuspidatum, *Sonchus* brachyotus and Scutellaria barbata. Its active pharmaceutical ingredients contain emodin, rhubarb anthraquinone, polydatin and flavonoids. A study has shown that RYN has an inhibitory effect on *S. aureus*, among which taraxacum inhibits the biofilm of *S. aureus* (Kenny et al., 2015). Antibiotics combined with RYN in the clinic are usually used to treat bacterial infections such as pneumonia and suppurative tonsillitis in China. The therapeutic effect and mechanism of the combination of RYN and antibiotics in the treatment of MRSA and its biofilm infections are unclear.

In the post-genome era, life science research has entered a comprehensive, systematic and dynamic functional exploration stage. The combined application of a variety of high-throughput research strategies, such as genomics, transcriptomics, proteomics and metabonomics, is more conducive to the comprehensive exploration of cell life processes. The selection of appropriate methods and tools will provide important support for the reliability and credibility of the research. In this study, real-time fluorescence polymerase chain reaction (RT–PCR) was used to analyze the gene expression of MRSA and its biofilm after treatment with LNZ combined with RYN (Rishi et al., 2018). Metabonomics is a technique to explore the changes in all endogenous small molecular metabolites (molecular weight less than 1500 Da) in cells after changes in external conditions (Emara et al., 2011). This has improved our understanding of the changes in intracellular metabolites. By identifying the metabolites of MRSA after drug intervention, we can further understand the effects of drugs on the molecular metabolism of bacteria from the planktonic state to the formation of biofilms. These small molecular metabolites are also likely to become therapeutic targets. In biological signalling, genes, as signal sources, are responsible for regulating and controlling the production of phenotypic metabolites. Changes in gene expression could affect the type and content of metabolites. The combination of gene detection and metabonomics technology provides technical support for exploring the mechanism of RYN combined with antibiotics against MRSA. In the present study, the synergistic effects of RYN and LNZ on MRSA and its biofilms were confirmed through bacteriostatic experiments and morphological observations *in vitro*. Then, to explore the mechanisms of these synergistic effects, the expression of MRSA biofilm-related genes was examined using RT–PCR, and the changes of endogenous metabolites of MRSA in mature biofilms were analysed by cell metabonomics technique. This study aims to clarify the synergistic inhibitory effects and corresponding mechanisms of RYN combined with LNZ on MRSA and its biofilm to lay a foundation for the establishment and optimization of an anti-MRSA regimen of RYN combined with LNZ, and to provide guidance for more safer and more effective clinical use.

## Materials and Methods

### Experimental Reagents and Bacterial Strains

The Reyanning mixture (RYN, Batch No. 180411) was obtained from Tsinghua Deren Xi’an Happiness Pharmaceutical Co., Ltd., China. According to 2020 China Pharmacopoeia, 1000 ml of RYN is from 372 g Taraxacum (Compositae; *Taraxacum mongolicum* Hand. -Mazz.), 372 g Polygonum cuspidatum (Polygonaceae; *Polygonum cuspidatum* Siebold and Zucc.), 372 g *Sonchus* brachyotus (Compositae; *Sonchus arvensis* L.), and 186 g Scutellaria barbata (Lamiaceae; *Scutellaria barbata* D. Don.). These herbs were decocted twice in water. The temperature of decoction was maintained at 96–100°C. The duration of decoction was 2 h for the first time and 1 h for the second time. The ratio of the herbs to solvent was 1:8 for the first time and 1:6 for the second time. Twice decocted solutions were mixed, centrifuged and filtered. Then the filtered solution was concentrated at 60–80°C, 0.01–0.09 Mpa steam pressure, and 0.04–0.08 Mpa vacuum degree. The concentration was stopped when the relative density reached 1.03–1.08 (70°C). Then 1.5 g stevioside and 0.5 g ethyl hydroxybenzene were added to the concentrated solution, and all were boiled and mixed well.

The product quality testing standards stipulate that the active ingredient content of the drug shall not be less than 0.15 mg/ml for emodin and 0.35 mg/ml for polydatin. The quality of RYN was controlled by chromatography, and no traces of heavy metals, organic solvents or other contaminants were found. The results showed that the two main components were emodin (C_15_H_10_O_5_, 0.95 mg/ml) and polydatin (C_20_H_22_O_8_, 2.44 mg/ml) (Supplementary Figures S1, S2; Supplementary Tables S1, S2).

Linedzolid injection (LNZ, Batch No. 14K03U03) was from Pfizer, Norway. Tryptone and yeast extracts were obtained from Oxoid, United Kingdom. NaCl from Merck, Germany, and 2,3,5-triphenyltetrazolium chloride (TTC) was obtained from Amresco, USA. 2,3-Bis-(2-methoxy-4-nitro-5-sulfophenyl)-2H-tetrazolium-5-carboxanilide (XTT) and phenazine methosulfate (PMS) were purchased from Sigma–Aldrich, USA. LIVE/DEAD ®BacLightTM Bacterial Viability Kit (Batch No. Lmuri 7012), FilmTracer™ SYPRO Ruby biofilm matrix stain (Batch No. 953539), and glutaraldehyde (Batch No. G6527) were purchased from Sigma–Aldrich, United States. Ethanol (Batch No. 10009527) and tert-butanol (Batch No. 80023928) were purchased from the Sinopharmaceutical Group, China. LC–MS grade acetonitrile and HPLC grade methanol were purchased from the Merck Company (Dannstadt, Germany), and formic acid (spectroscopic grade) was purchased from the CNW Company. The experimental water was Watsons distilled water. All chemical standards were purchased from Sigma–Aldrich (MO, United States) unless otherwise specified.

MRSA was extracted and isolated from the sputum of inpatients in the Respiratory Department of Beijing Dongzhimen Hospital and maintained in cryogenic storage at −80°C on glass beads. The experimental strain was continuously cultured for 10 generations in the laboratory and repeatedly induced by related antibiotics for five generations, and there was no significant difference in MICs before and after the two, which proved that it had genetic stability. The working culture of the bacteria was maintained on an agar plate at 4°C and subcultured in Luria-Bertani (LB) broth (10 g of tryptone, 5 g of yeast extract, and 10 g of NaCl/litre) before use. In short, a single MRSA colony was inoculated into LB culture medium and incubated with 280 rpm at 37°C for 24 h. The broth culture overnight was diluted with fresh medium to obtain an initial inoculation size of OD600 = 0.02.

### Determination of the MIC

MICs of RYN and LNZ were determined by a standard broth microdilution method in sterile 96-well microplates (Standards, N.C.F.C.L, 2000). Two-fold serial dilutions were made in LB broth over a range to give final concentrations of 1/2–1/1024 for RYN solution and 32–0.0625 μg/ml for LNZ. Then 100 μl of bacterial suspension (OD600 = 0.02) was added to each well.

The negative control was comprised of LB broth and the tested sample while the positive control was LB broth and bacterial suspension only. The final volume of each well was 200 μl. The MICs of the test samples were detected after 24 h of incubation at 37°C, followed by the addition of 30 μl of 2.5 mg/ml TTC and incubation for an additional 20 min at 37°C (Yang et al., 2018). Viable bacteria reduced the yellow dye to pink. The MIC was defined as the lowest sample concentration that prevented this change and exhibited complete inhibition of microbial growth (Fankam et al., 2011).

### Checkerboard Assay

The combined effect of RYN with LNZ was evaluated by the checkerboard microdilution method, and the fractional inhibition concentration index (FICI) of the drug-drug interaction was obtained (Basri and Sandra, 2016). The concentration range of multiple dilutions was set according to the single use MIC of the drug (LNZ: 4–0.0625 μg/ml, RYN: 1/4–1/256RYN solution). Then, 100 μl of drug solution and 100 μl of bacterial suspension (OD600 = 0.02) were added to each well. After 24 h of incubation at 37°C, 40 μl of TTC was added to each well, and the plates were incubated again for 20 min. Wells containing the solution that turned pink comparable to that of the positive control were interpreted as positive for bacterial growth, while the wells containing colourless solutions were interpreted as negative for bacterial growth. The FICI evaluated the bacteriostatic effect of the combination of the two drugs: FICI= (A/MICA) + (B/MICB). A and B are the respective MICs of drugs in combined bacteriostasis. MICA and MICB drugs were used alone with the bacteriostatic MIC. The results were interpreted as synergy (FICI≤0.5), addition (0.5 < FICI≤1), indifference (1 < FICI≤2) or antagonism (FICI>2) (Yu et al., 2005). The experiments were performed in triplicate and the median FICI values were used in the analysis.

### Biofilm Assay

The XTT reduction assay is considered to be proportional to the metabolic activity of cells (Kannappan et al., 2017). The bacterial liquid was diluted to OD600 = 0.1 with LB medium containing 0.25% glucose (LB-G) and incubated at 37°C for 0 and 24 h. The corresponding concentrations of RYN and LNZ were prepared. The concentration of RYN is 1/4MIC (1/8RYN), 1/8MIC (1/16RYN), 1/16MIC (1/32RYN) and 1/32MIC (1/64RYN), while that of LNZ is 1/2MIC (2 μg/ml). The same amount of drug solution (100 μl) as the bacterial solution was added to the 96-well plate of the mature biofilm. After incubation at 37°C for 24 h, the planktonic cells were washed out. Forty microlitres of XTT-PMS solution was added to each well and incubated in the dark at 37°C for 20 min. The optical density was measured by an enzyme-labelled instrument at 450 and 655 nm.

### Biofilm Determination by Confocal Laser Scanning Microscopy

Mature biofilms of MRSA were cultured according to our previous study (Yang et al., 2018) The structural parameters of individual cellular and extracellular components of biofilms formed by MRSA strains were evaluated by CLSM observations and digital image analysis (Cerca et al., 2012). Briefly, the biofilm was washed twice with LB-G after being exposed to RYN and LNZ for 24 h. The viability of cells in the biofilm was determined by SYTO9/PI staining, and the extracellular matrix of the biofilm was determined by Matrix dye. The quartile glass Petri dish was then incubated in the dark at 37°C for 20 min. After washing the dye with LB-G medium, 500–600 μl LB-G medium was added to each well to keep the well moist. CLSM image acquisition was performed using Olympus TM FluoView FV1000 (Olympus, Lisboa, Portugal) confocal scanning laser microscope. Three stacks of horizontal plane images (1024 × 1024 pixels) with a z-step of 5 µm were acquired for each well from three different randomly chosen areas (Gomes et al., 2011). For image analysis, original Olympus files (OIB format) were imported into the IMARIS 9.1 software package (Bitplane, Zurich, Switzerland). The individual components of biofilms were represented by fluorescence emitted by SYTO9 (from cells with live membranes), PI (from bacteria with dead membranes) and Matrix (from bacteria with extracellular matrix). Three independent experiments were performed for each MRSA strain.

### Scanning Electron Microscope

MRSA mature biofilms were cultured on 5 × 5 mm diameter round basic slides (Agar Scientific, Stansted, United Kingdom) in the wells of a 24-well plate (Wang et al., 2011). The unbound suspension bacteria were discarded, and 2 μg/ml LNZ, 1/16RYN and 2 μg/ml LNZ+1/16RYN were added. Blank LB-G medium of the same volume was added to the control group. The plate was exposed to a box with constant temperature and humidity at 37°C for 24 h. The slides were then stabilized in 1 ml of 4% glutaraldehyde for 1 h, fixed with 4% osmium acid, and dehydrated in serial dilutions (50, 70, 90, 100% v/v) of ethanol for 20 min. Subsequently, the slides were immersed in tertbutyl alcohol for 1 h. The slides were air-dried for 2 days and then transferred to copper disks and dusted with gold (160 s, 40 mA). The samples were analysed under SEM (JEOL JSM-35CF, SEM, JEOL, Japan) under 20–25 kV voltage (Absalon et al., 2011; Krzysciak et al., 2017).

### Permeability Assay

The effects of RYN and LNZ on the structure of biofilms were studied by simulating an experimental model of antibiotic permeation *in vitro* (Anderl et al., 2000). In a 6 cm Petri dish, 4 ml of bacterial liquid (OD600 = 0.1) and 4 ml of LB-G were added, the blue filter membrane was put into the bacterial solution and cultured in a constant temperature incubator at 37°C for 48 h, and a mature biofilm was formed on the filter membrane. The mature biofilm was supplemented with 2 μg/ml LNZ, 1/16RYN and 2 μg/ml LNZ+1/16RYN, and the control group was supplemented with the same volume of blank LB-G. Then the 6 cm Petri dish was placed in a constant temperature incubator and cultured at 37°C for 24 h. The MRSA biofilm was transferred from 6 cm plate to a plate containing vancomycin (500 μg/ml). The bacterial biofilm was covered with a filter membrane to form a bacterial biofilm sandwich, and 6 mm filter paper was placed on the sandwich structure. After 2, 4 and 6 h, the filter paper was removed and affixed to a plate coated with MRSA. The plate was placed in a constant temperature incubator and cultured at 37°C for 18 h, and the bacteriostatic zone was observed.

### Detection of the Expression of MRSA Biofilm Related Genes by RT–PCR

#### Extraction of Total RNA

The preparation of total RNA from MRSA was performed using RNA protection reagent according to the manufacturer’s instructions (TIANGEN, China). Briefly, total RNA was prepared by lysostaphin extraction using OD600 = 0.1 of bacteria at 24 h, followed by further purification with an RNA prep Pure Cell/Bacteria Kit (TIANGEN, China) according to the manufacturer’s instructions. The quality and quantity of total RNA were confirmed by agarose electrophoresis and UV spectrophotometry, respectively.

#### RT–PCR

Contaminating chromosomal DNA was removed by DNase treatment (TaKaRa, Japan). Purified *S. aureus* RNA was reverse transcribed into cDNA by the PrimeScript™ RT reagent Kit with gDNA Eraser (TaKaRa, Japan) and then subjected to RT–PCR analysis using an ABI 7500 thermocycler (Applied Biosystems, America). The relative quantification of MRSA strain scripts was determined by the ratio of expression of target transcripts relative to *agrA*, *agrB*, *agrC*, *agrD*, *atlA*, *RNAⅢ*, and *sarA*. The sequences of primers for RT–PCR experiments are provided in Table 1.

**TABLE 1:** Primers used in this study.

Primer name	Primer sequence (5′to3′)	Product size(bp)
*agrA*-F	TGT​TAT​CAA​TGG​TCA​CTT​ATG​CTG	323
*agrA*-R	GTT​TGC​TTC​AGT​GAT​TCG​TTT​ATT	
*agrB*-F	AGT​ACG​TTT​AGG​GAT​GCA​GGT​C	176
*agrB*-R	CCA​CAT​AAC​ACC​AAA​ATG​AAG​AAG	
*agrC*-F	CTT​GAT​AAC​GCA​ATA​GAG​GCA	179
*agrC*-R	CCT​AAA​CCA​CGA​CCT​TCA​CC	
*agrD*-F	TCA​TTT​TTT​GAT​TTT​ATA​ACT​GGT​G	101
*agrD*-R	TCT​TTA​GGT​ATT​TCA​ACT​TCG​TCC	
*atlA*-F	ACG​TGT​ACC​AGG​TAA​GTG​GAC​AGA	191
*atlA*-R	AAT​GCT​GGA​TCT​TGA​GCT​AAA​CG	
*RNAⅢ*-F	TAA​ACA​TCC​CAA​CTT​GCC​AGA	205
*RNAⅢ*-R	ATC​CAA​ATA​CAA​TGC​CCC​AAT	
*sarA*-F	ATG​GGG​AAC​ATG​ATC​CTT​TG	294
*sarA*-R	TAG​CCG​CAT​AAC​GAG​CAG​TA	
16s rRNA-F	TTC​TGG​TCT​GTA​ACT​GAC​GCT​G	299
16s rRNA-R	CGA​AGG​GGA​AGG​CTC​TAT​CT	

### UPLC-TOF-MS Detection of Metabolites in MRSA Biofilm

#### Metabolite Extraction

Intracellular metabolites from each biological replicate were extracted using a cold methanol extraction approach as previously reported (Zhu et al., 2014). MRSA bacteria were collected at 0 and 24 h after administration. To put it simply, 60% methanol water was placed in a refrigerator at −20°C for pre-cooling. The precultured bacterial liquid was loaded into a 15 ml centrifuge tube, and each tube was 7 ml. Methanol water was added to the centrifuge tube at a ratio of bacterial liquid: methanol water = 1:1, shaken by hand for 5 s, and then placed on the ice in a refrigerator. The bacterial solution was centrifugally quenched at 3000 rpm for 10 min at 4°C, and the supernatant was removed after centrifugation. The protein was precipitated by adding methanol to the sample, fully vortexed for 30 s and then centrifuged at 4°C 120000 rpm for 15 min, and the supernatant was extracted for detection.

#### Metabolite Analysis

Ultra-high-pressure liquid chromatography-time-of-flight mass spectrometry (UPLC-Q-TOF-MS) was described elsewhere and was used with minor modifications (Huang et al., 2013). Briefly, a 4 μl aliquot was chromatographed using an Agilent C18 analytical column (i.d. 100 × 2.1 mm, particle size 1.8 μm). Mobile phase A and mobile phase B were water/formic acid (99.9:0.1, v/v) and acetonitrile/formic acid (99.9:0.1, v/v), respectively, and the flow rate was 0.4 ml/min. The column eluent was directed to the mass spectrometer for analyses. When a Premier mass spectrometer operating in the positive ion electrospray mode was used, the instrumental parameters were set as follows: the capillary voltage was set to 3.0 kV; the sampling cone voltage was set to 35.0 V; the nitrogen drying gas was set at a constant flow rate of 600 L/h; the source temperature was 100°C; the desolvation temperature was 350°C; the cone gas flow was 50 L/h; and the extraction cone was 4 V. When a Premier mass spectrometer operating in the negative ion electrospray mode was used, the instrumental parameters were set as follows: the capillary voltage was set to 3.5 kV; the sampling cone voltage was set to 50 V; the nitrogen drying gas was set at a constant flow rate of 600 L/h; the source temperature was 100°C; the desolvation temperature was 350°C; the cone gas flow was 50 L/h; and the extraction cone was 4 V. MS/MS analysis was performed on the mass spectrometer set at different collision energies according to the stability of each metabolite. The time-of-flight analyser was used in the V mode and was tuned for maximum resolution (>10,000 resolving power, atm/z556.2771). The instrument was previously calibrated with sodium formate; the lock mass spray for precise mass determination was set by leucine enkephalin atm/z 556.2771 with a concentration of 0.5 ng/μL. Fifteen injections of QC samples were performed to equilibrate the UPLC–MS system before running the actual samples. QC samples were injected every six samples at regular intervals throughout the analytical run.

#### Data Processing and Statistical Analysis

These data were preprocessed by Mass Profiler software (Agilent) and edited later in EXCEL2007 software. The final result is organized into a two-dimensional data matrix, including variables (retention time and mass-charge ratio), observation (sample) and peak intensity. The sample of this project obtained 6623 features in positive mode and 3581 features in the negative mode. All of the data are then normalized to the total signal integral. The edited data matrix was imported into SIMCA-P software (Umetrics AB, Umea, Sweden, version 13.0) for principal component analysis (PCA). The SAS statistical package (order no. 195557), version 9.1.3, was used for the statistical analysis. The attribute data were analysed using the χ-square test. The measurement data obtained indicated a normal distribution. Comparisons between multiple groups were analysed using analysis of variance. Metabolites with significant differences were screened by the VIP (VIP>1), *p* value (*p* < 0.01) and fold change value (FC < -3, FC > 3). Metabolic pathway analysis was performed using MetaboAnalyst software (version 3.0). The calculated *p* value was established on the basis of the pathway enrichment analysis whereas the pathway impact value was derived from the pathway topology analysis. Correlation analysis using was performed using the Pearson correlation coefficient with R (pheatmap package) software.

## Results

### MICs and the MIC Ratio of LNZ and RYN to MRSA

The MICs of LNZ and RYN to MRSA were 4 μg/ml (MICa) and 1/2RYN (MICb) solutions respectively, and the initial concentration of the chessboard method was determined accordingly (Table 2). When 2 μg/ml LNZ (1/2MICa)+1/32RYN (1/16MICb) was used as the MIC combination of MRSA (FICI = 0.625), the combination of LNZ and RYN showed an additive effect (Table 2). The experimental results show that the combination of RYN and LNZ can reduce the concentration of LNZ against MRSA (Supplementary Figure S3).

**TABLE 2:** The MICs, FICI and action mode of LNZ and RYN on MRSA.

Strain	LNZ (μg/ml)	RYN			FICI	Outcome
	Alone	Combination	Alone	Combination		
MRSA	4	2	1/2	1/32	0.625	Addition

### Effects of Single and Combined Use of LNZ and RYN on Living Bacteria in MRSA Biofilms

#### Effects of LNZ and RYN on Living Bacteria in Biofilms at the Mature Stage of MRSA

**FIGURE 1:**
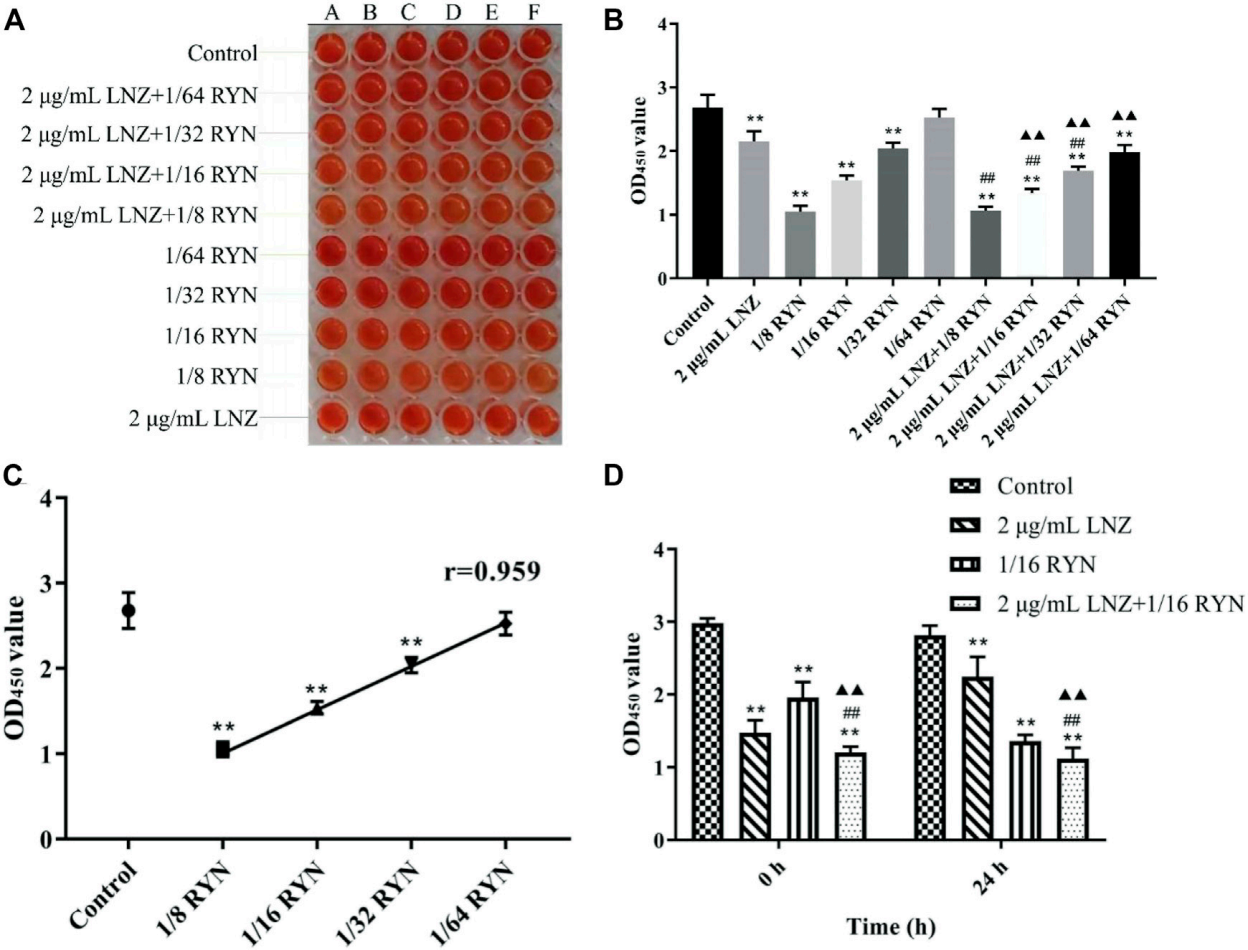
Effects of single and combined use of LNZ and RYN on living bacteria before and after MRSA biofilm production. (**p < 0.01 vs. control group; ^##^ *p* < 0.01 *vs*. 2 μg/ml LNZ group; ^▲▲^ *p* < 0.01 *vs*. 1/16RYN group.). **(A)** Results of XTT staining; **(B)** The effects of single and pairwise use of LNZ of 1/2 MIC and four doses of RYN on the number of living bacteria in MRSA biofilms; **(C)** The effect of RYN on the number of living bacteria in MRSA biofilm was dose-dependent; **(D)** Before and after the formation of biofilms, the effect of 2 μg/ml LNZ+1/16RYN was better than that of the two drugs.

As shown in Figures 1A–C, when MRSA biofilms matured, except that 1/64RYN had no obvious inhibitory effect on the living bacteria in MRSA biofilms, 1/8RYN, 1/16RYN and 1/32RYN all had bacteriostatic effects in a dose-dependent manner. The r value of linear regression analysis is 0.959. LNZ (2 μg/ml) inhibited the living bacteria in MRSA biofilms, but the inhibitory effect was not as good as that of the 1/8RYN group and 1/16RYN group. Viable bacteria in MRSA biofilms were inhibited in the positive drug LNZ group, but the inhibitory effect was not as good as that the in 1/8RYN group and 1/16RYN group. It is suggested that both RYN and LNZ can destroy the structure of MRSA biofilm and infiltrate into the biofilm to kill the deep bacteria in the biofilm, but the effect of LNZ is weaker than that of high and middle dose of RYN.

As shown in Figure 1B, the effect of the combination of LNZ and RYN is better than respective those of the two drugs alone, and the combination significantly affected the change in living bacteria in biofilms. The combination effect is improved with increasing RYN concentration and dose. There were significant differences between 2 μg/ml LNZ+1/16RYN group and the 2 μg/ml LNZ group and the 1/16RYN group, and the effect was better than that of 2 μg/ml LNZ+1/32RYN group. This experiment shows that the best combined dose of RYN combined with LNZ is 2 μg/ml LNZ+1/16RYN.

#### Effects of LNZ and RYN on Living MRSA Before and After the Biofilm Formation

As shown in Figure 1D, there was a significant difference in the amount of live MRSA bacteria in the effect of drugs before and after biofilm formation. Compared with control group, 2 μg/ml LNZ had a better inhibitory effect on MRSA living bacteria before biofilm formation than 1/16RYN at 0 h. In the case of the combination of the two drugs, the inhibitory effect of the 2 μg/ml LNZ+1/16RYN group on living MRSA before and after biofilm formation was greater than that of the two drugs alone. The results show that the combination of LNZ and RYN has a significant inhibitory effect on living MRSA before and after biofilm formation.

### CLSM Observation of the Effects of Drugs on MRSA Biofilm in Living Bacteria, Dead Bacteria and Extracellular Matrix

**FIGURE 2:**
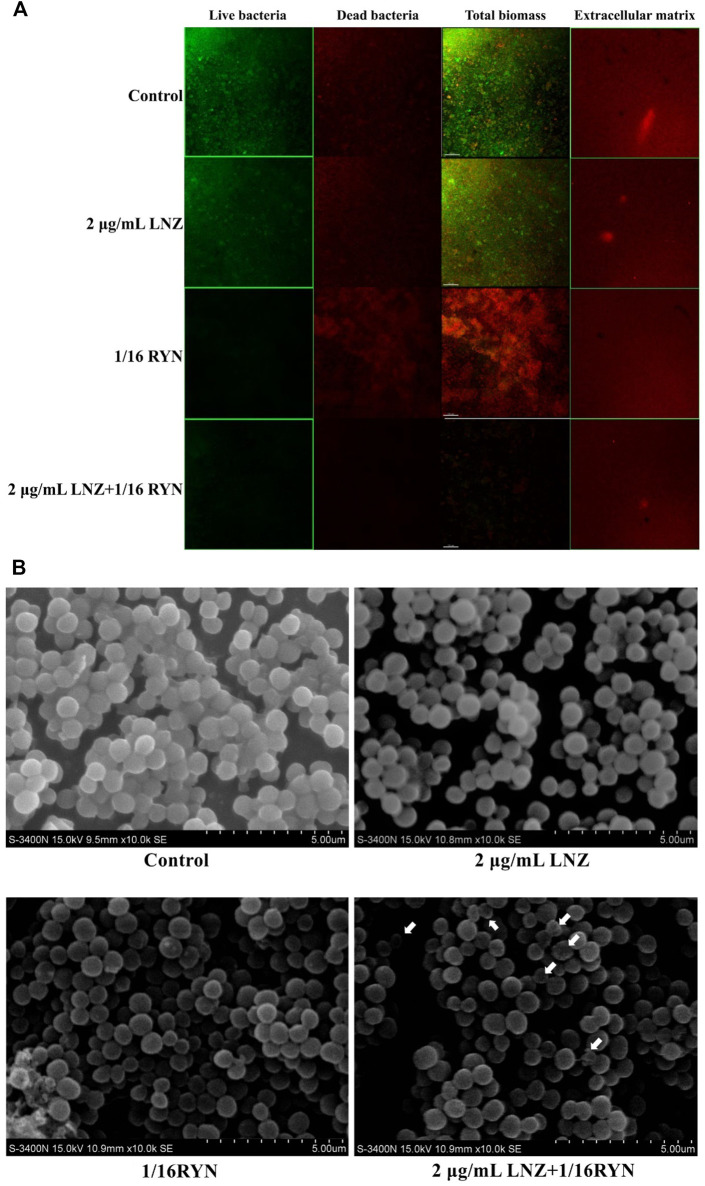
Imaging results of the effects of LNZ and RYN on MRSA biofilm live and dead bacteria and extracellular matrix. **(A)** CLSM. In the first three columns, green is the fluorescence signal of living bacteria, and red is the fluorescence signal of dead bacteria. The images show the inhibitory effect of LNZ and RYN on the maturity of MRSA biofilms, which is consistent with the results of the biofilm assay, and the combined group had a strong inhibitory effect on biofilms. There was no significant difference among the four groups in the extracellular matrix of the fourth column. The scale bar in images represents 150 µm. **(B)** SEM. In the mature stage of a MRSA biofilms without drug intervention (control group), MRSA grew in clumps and the structure of the cell arrangement was compact. In the mature stage of MRSA biofilms treated with 2 μg/ml LNZ and 1/16RYN, the structure of the cell arrangement was slightly loose. Combined 2 μg/ml LNZ+1/16RYN intervention in the mature stage of a MRSA biofilm resulted in a loose cell arrangement structure, a different cell size, and an empty shell. Original magnification: ×10000.

As shown in Figure 2A, the surface of the control group was rough, the fluorescence signal value of living bacteria was strong, the vitality of living bacteria in biofilms was strong, the distribution of bacteria was tight, and the fluorescence signal value of dead bacteria is weaker than that of living bacteria. Compared with the control group, the light spot of living bacteria in the 2 μg/ml LNZ group decreased slightly due to drug action, but the signal intensity was still larger, and the fluorescence signal values of dead bacteria in the two groups were similar. However, the fluorescence signal of living bacteria was significantly weakened and the fluorescence signal of dead bacteria in the 1/16RYN group was significantly stronger than those in the former two groups. The fluorescence signal values of living and dead bacteria in the combined group (2 μg/ml LNZ+1/16RYN) were significantly lower than those in the first three groups. Its surface is finer than that of other groups, the thickness of biofilm decreases obviously, and its distribution is sparse. The administration group did not have any inhibitory effect on the extracellular matrix of MRSA biofilms. These results suggested that RYN could destroy the protective effect of biofilms on the bacteria itself and that combination with LNZ could inhibit and kill bacteria in biofilms. It has not only inhibition and killing effects on living bacteria, but also scavenging effects on the residual structure of dead bacteria.

### Effect of LNZ and RYN on MRSA Biofilm by SEM

As shown in Figure 2B, the bacteria gathered on the MRSA biofilm surface of the control group were wrapped in a membranous structure, and the bacteria in the biofilm were uniform in size and normal in shape. There was a membrane structure in the MRSA of the positive drug group (2 μg/ml LNZ), and there was no significant difference in the size of bacteria between the 2 μg/ml LNZ group and the control group. In the 1/16RYN group, there was no obvious membrane structure in the outer layer of MRSA, the arrangement of bacteria was no longer compact, and the shape of bacteria was irregular and round. In the 2 μg/ml LNZ+1/16RYN group, there was no membrane structure in the outer layer of MRSA, the arrangement structure of bacteria was loose, the size of bacteria was different, and there were bacteria in the empty shell state (shown by white arrow). It is suggested that RYN has a destructive effect on MRSA biofilms, and the combination of the two drugs can increase the inhibitory effect on bacteria in the mature stage of MRSA biofilms.

### Effect of LNZ and RYN on the Permeability of MRSA Biofilm

**FIGURE 3:**
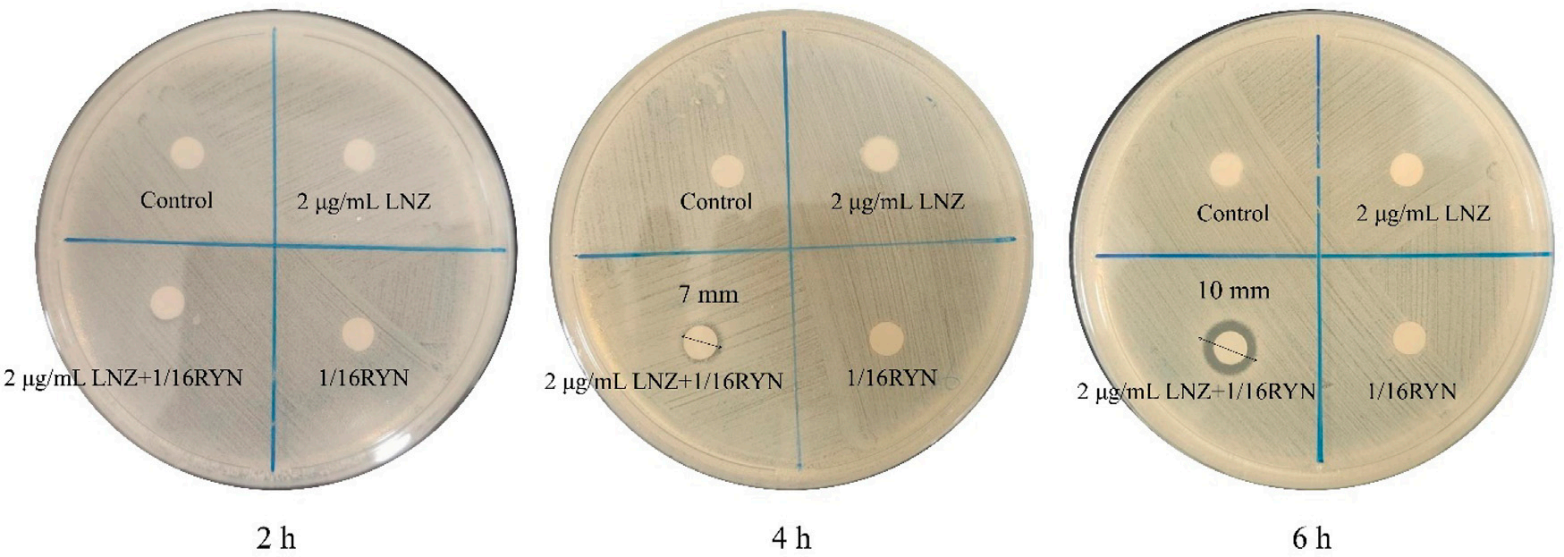
Effect of LNZ and RYN on the permeability of MRSA biofilms. There was no bacteriostatic zone in the control group, 2 μg/ml LNZ group and 1/16RYN group, but the bacteriostatic zone in the 2 μg/ml LNZ+1/16RYN group increased with the extension of action time [2 h (6 mm) < 4 h (7 mm) < 6 h (10 mm)].

As shown in Figure 3, there is no bacteriostatic zone in the control group, 2 μg/ml LNZ group and 1/16RYN group at 2, 4 and 6 h, while in the 2 μg/ml LNZ+1/16RYN group, there is a bacteriostatic zone at 4 and 6 h, and the size of bacteriostatic zone was 2 h (6 mm) < 4 h (7 mm) <6 h (10 mm). The results show that the combination of LNZ and RYN has an effect on the structure of the MRSA biofilm, and the permeability of the MRSA biofilm is improved compared with the single drug group.

### Detection Results of MRSA Biofilm Related Genes by RT–PCR

The amplification curves of *agrA*, *agrB*, *agrC*, *agrD*, *atlA*, *RNAⅢ* and *sarA* were consistent, and the dissolution curves were all single peaks, indicating that the specificity of each gene primer was strong, and that the amplification product was single (Supplementary Figure S4).

**FIGURE 4:**
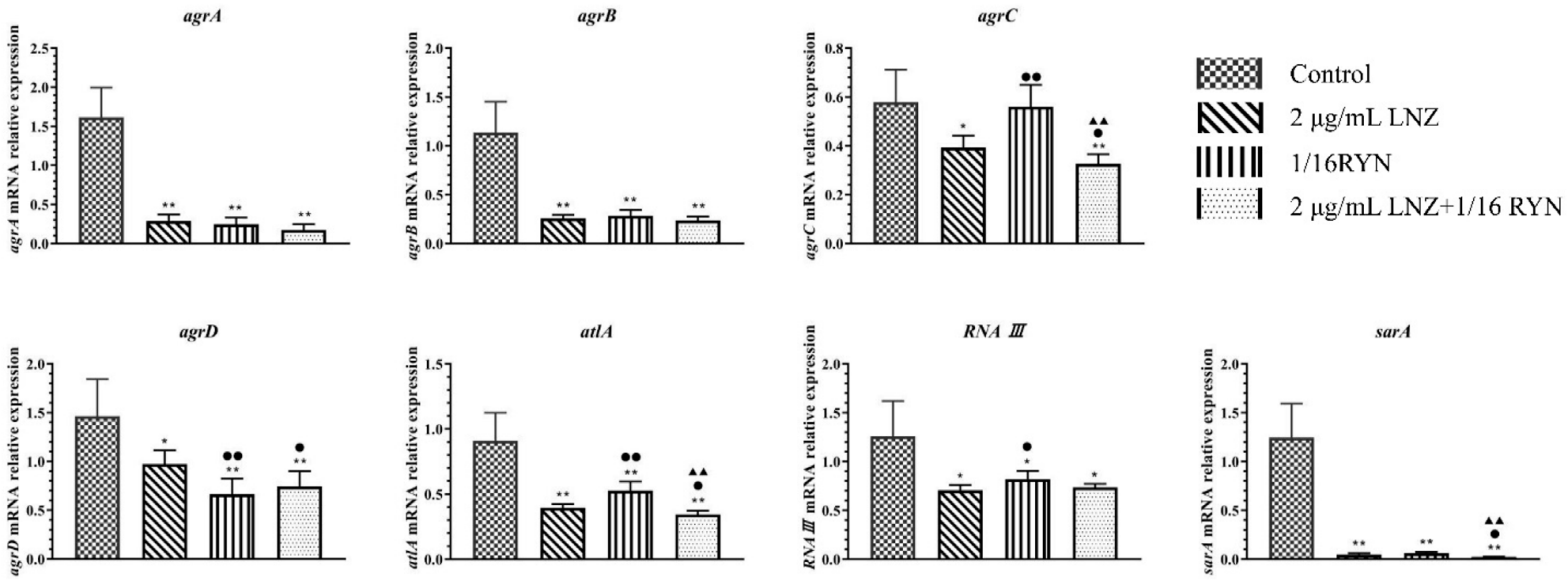
mRNA expression of biofilm-related genes after different drug treatments. (**p < 0.01, *p < 0.05 *vs*. control group; ^▲▲^ *p* < 0.01, ^▲^ *p* < 0.05 *vs.* 1/16RYN group; ^●●^ *p* < 0.01, ^●^ *p* < 0.05 *vs.* 2 μg/ml LNZ group.). Compared with the control group, the gene expression decreased after drug intervention. The expression of *agrC*, *atlA* and *sarA* decreased significantly compared with LNZ and RYN alone under the intervention of the combination of the two drugs.

The expression of the MRSA biofilm-related genes *agrA*, *agrB*, *agrC*, *agrD*, *atlA*, *RNAⅢ* and *sarA* was inhibited after drug intervention in the formation of biofilms (Figure 4). Compared with the control group, the expression of *agrA*, *agrB*, *agrC*, *agrD*, *atlA*, *RNAⅢ* and *sarA* was inhibited in the 2 μg/ml LNZ group and the 2 μg/ml LNZ+1/16RYN group. In the 1/16RYN group, the expression of six genes except for *agrC* was downregulated. In the comparison of RYN and LNZ alone, the inhibitory effect of *agrD* in the 1/16RYN group was higher than that in the 2 μg/ml LNZ group. In the comparison of drug use alone and in combination, the inhibitory effect of *agrC*, *agrD*, *atlA* and *sarA* in the 2 μg/ml LNZ+1/16RYN group is higher than that in the 2 μg/ml LNZ group, and the inhibitory effect of *agrC*, *atlA* and *sarA* is higher than that in the 1/16RYN group.

### Screening Differential Metabolites of RYN and LNZ Against MRSA Biofilms

**FIGURE 5:**
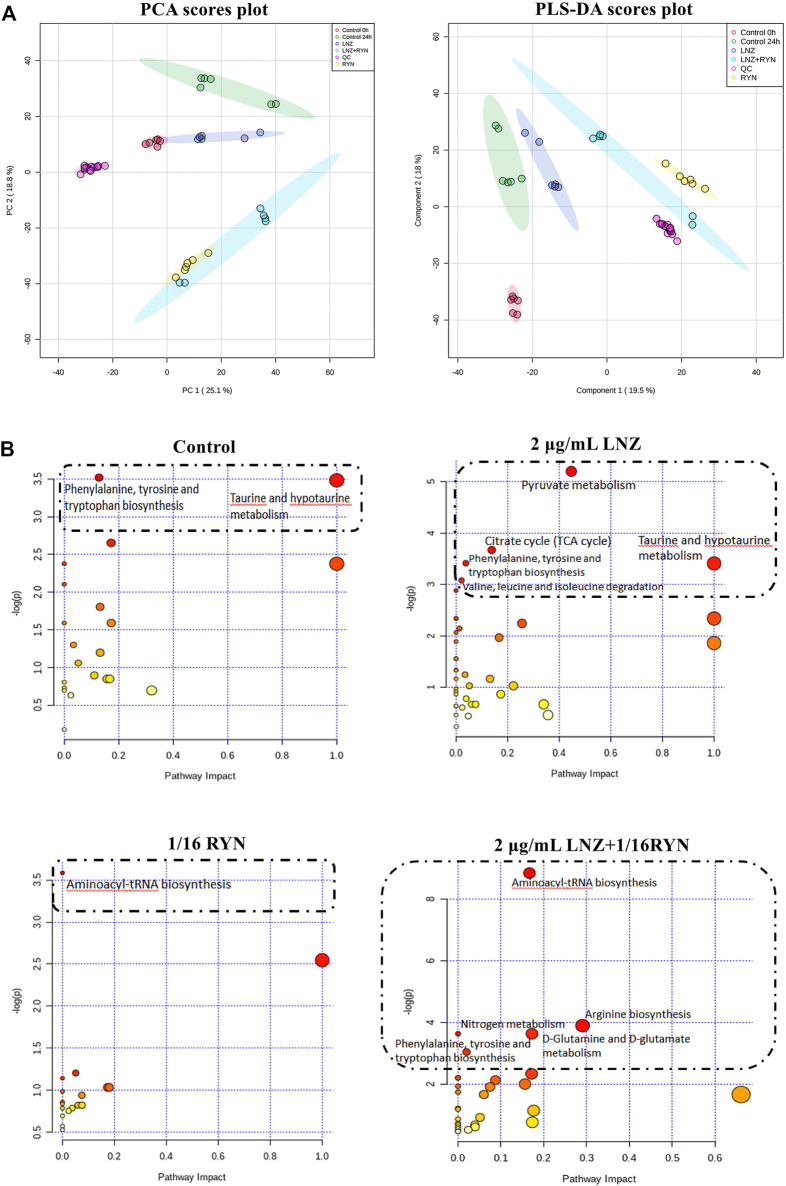
PCA and PLS-DA score plots and overview of pathway analysis metabolites. **(A)** PCA and PLS-DA score plots. PCA score plot of all samples including QC samples. PLS-DA score plot of all samples including QC samples. **(B)** overview of pathway analysis metabolites. The bubble chart of pathway enrichment analysis shows all matched pathways according to the path impact values (*X*-axis) of topological analysis and *p* value (*Y*-axis) of path enrichment analysis. Each node represents a biological pathway. Bubble area (radius) is proportional to the impact value of each pathway, with the colour denoting the significance from highest in red to lowest in white. The nodes in the dashed box represent the biological pathways that are significantly related to biofilm markers.

UPLC-Q-TOF-MS analysis of the MRSA samples from the biofilm period indicated the presence of 6623 metabolite features in the positive mode and 3581 metabolite features in the negative mode, of which 2181 were putatively identified. The PCA scores of all metabolites in the six groups are shown in Figure 5A. The dataset was processed via an unsupervised statistical approach using PCA. PCA was used to investigate any subdata clustering, which was not evident. Furthermore, the dataset was assessed for outliers via a distance of observation (DModX) analysis, which indicated that no samples exceeded the threshold for rejecting a sample. However, some RYN and LNZ + RYN samples were intermixed with each other, although there was a tendency for the samples to be separated between the two groups. To further validate that the metabolic differences between MRSA were the result of metabolic consequences induced by LNZ, PLS-DA was used to compare the metabolic profiles of the three groups. Five groups of samples and QC could be separated in the PLS-DA score plot (Figure 5A).

Twenty-seven differential metabolites were found in the comparison of the two control groups before and after biofilm formation (Table 3). These metabolites reflect the metabolic phenotype characteristics of MRSA biofilm formation, and are significantly related to taurine and hypotaurine metabolism and phenylalanine, tyrosine and tryptophan biosynthesis (Figure 5B, Supplementary Table S3).

**TABLE 3:** Different metabolites in each group.

Compound	R.T (min)	Exact mass	Formula	Fold change			
				Control	2 μg/ml LNZ	1/16RYN	2 μg/ml LNZ+1/16RYN
1,3,7-trimethyluric acid	5.96	210.075	C_8_H_10_N_4_O_3_	5.925	—	—	—
1-O-caffeoylglucose	1.98	342.095	C_15_H_18_O_9_	—	—	—	−5.034
2-methylbutanoyl-coenzyme A	5.36	851.173	C_26_H_44_N_7_O_17_P_3_S	7.369	5.391	—	−4.962
2-succinylbenzoyl-coenzyme A	1.09	971.158	C_32_H_44_N_7_O_20_P_3_S	−7.372	5.004	—	—
3-dehydrocarnitine*	4.74	160.097	C_7_H_13_NO_3_	−9.932	5.758	5.307	12.805
acetyl phosphate	0.9	139.988	C_2_H_5_O_5_P	10.32	−4.781	—	—
acetyl-coenzyme A	3.81	809.126	C_23_H_38_N_7_O_17_P_3_S	—	3.86	—	—
ADP-D-ribose*	0.67	559.072	C_15_H_23_N_5_O_14_P_2_	−5.387	−11.314	4.462	4.324
anthranilic acid	0.79	137.048	C_7_H_7_NO_2_	6.859	—	—	—
bis (3′,5′)-cyclic diguanylic acid	1.26	690.095	C_20_H_24_N_10_O_14_P_2_	—	—	3.652	—
citrulline	6.36	175.096	C_6_H_13_N_3_O_3_	—	—	−5.688	−5.667
cyclic AMP	1.75	329.053	C_10_H_12_N_5_O_6_P	4.493	—	—	−4.947
cytidine	1.1	243.086	C_9_H_13_N_3_O_5_	—	6.889	—	—
cytosine	0.84	111.043	C_4_H_5_N_3_O	−6.288	—	3.139	—
*D*-alanine	1.1	89.048	C_3_H_7_NO_2_	—	−4.322	—	—
D-butyrine	0.73	103.063	C_4_H_9_NO_2_	−4.792	—	—	—
dCMP	0.71	307.057	C_9_H_14_N_3_O_7_P	6.882	—	—	—
dephospho-coenzyme A	3.64	687.149	C_21_H_35_N_7_O_13_P_2_S	—	4.549	12.092	—
*D*-erythro-dihydrosphingosine	10.32	301.298	C_18_H_39_NO_2_	—	6.844	—	—
D-lactic acid	0.72	90.032	C_3_H_6_O_3_	4.197	−4.223	—	—
dTMP	0.89	322.057	C_10_H_15_N_2_O_8_P	—	—	—	3.784
gamma-glutamyl phosphate	6.79	227.02	C_5_H_10_NO_7_P	—	-3.496	—	—
glycerol	9.7	92.047	C_3_H_8_O_3_	—	—	—	3.256
glycocholic acid	8.86	465.309	C_26_H_43_NO_6_	—	—	4.7	—
histamine	0.76	111.08	C_5_H_9_N_3_	−3.219	—	—	—
homocitrulline*	6.99	189.111	C_7_H_15_N_3_O_3_	3.616	−3.132	−3.955	−3.869
inosine	7.26	268.081	C_10_H_12_N_4_O_5_	—	−5.497	−4.391	-
kynurenine*	2.08	208.085	C_10_H_12_N_2_O_3_	3.41	−3.112	−3.555	−5.055
L-carnitine	0.77	161.105	C_7_H_15_NO_3_	−4.065	—	—	—
*L*-cystathionine	0.7	222.067	C_7_H_14_N_2_O_4_S	—	—	—	4.573
L-glutamic acid	0.73	147.053	C_5_H_9_NO_4_	−7.229	—	—	6.018
*L*-glutamine	2.27	146.069	C_5_H_10_N_2_O_3_	—	−3.267	-	−6.258
*L*-histidine	0.81	155.07	C_6_H_9_N_3_O_2_	—	—	−5.45	−5.502
*L*-leucine*	1.09	131.095	C_6_H_13_NO_2_	0.377	−10.794	−6.502	−14.485
*L*-lysine*	0.77	146.106	C_6_H_14_N_2_O_2_	0.531	−3.684	−4.973	−7.645
*L*-malic acid	0.83	134.022	C_4_H_6_O_5_	—	13.672	—	—
*L*-phenylalanine	3.74	165.079	C_9_H_11_NO_2_	—	—	—	−3.613
*L*-proline	0.78	115.063	C_5_H_9_NO_2_	—	—	4.285	4.521
L-sorbose	0.76	180.063	C_7_H_14_O_5_	3.127	—	−6.449	—
*L*-tryptophan	8.89	204.09	C_11_H_12_N_2_O_2_	—	−3.136	—	−4.905
L-tyrosine	1.13	181.074	C_9_H_11_NO_3_	5.083	−5.689	—	−9.918
L-urobilin	4.7	594.342	C_33_H_46_N_4_O_6_	3.156	—	—	—
N-acetyl-D-phenylalanine	5.54	207.09	C_11_H_13_NO_3_	4.615	—	−5.425	—
phenylpyruvic acid	1.14	164.047	C_9_H_8_O_3_	7.009	3.852	5.721	—
phosphorylcholine	3.71	169.05	C_4_H_12_NO_4_P	—	—	—	−4.34
pyrrolidonecarboxylic acid	0.85	129.043	C_5_H_7_NO_3_	−7.133	—	—	5.791
ribose	0.73	150.053	C_5_H_10_O_5_	—	—	4.481	4.761
S-adenosylmethionine	7.24	398.137	C_15_H_22_N_6_O_5_S	—	—	8.838	7.276
sebacic acid*	0.9	202.121	C_10_H_18_O_4_	3.932	9.14	−4.511	−9.166
sn-glycerol-3-phosphate*	0.7	172.014	C_3_H_9_O_6_P	4.181	−3.816	−10.373	−9.086
sphingosine-1-phosphate*	1.95	379.249	C_18_H_38_NO_5_P	2.548	−10.714	−10.876	−8.764
succinic acid	1.14	118.027	C_4_H_6_O_4_	—	7.623	—	13.879
sucrose*	1.07	342.116	C_12_H_22_O_11_	6.765	−3.593	3.954	3.546
taurolithocholate-3-sulfate	4.29	563.259	C_26_H_45_NO_8_S_2_	—	—	—	−7.35
*trans*-oct-2-enoyl-coenzyme A	1.1	891.204	C_29_H_48_N_7_O_17_P_3_S	−6.914	—	—	—
tyrosine methyl ester	10.24	195.09	C_10_H_13_NO_3_	—	—	-	4.83
uric acid	0.98	168.028	C_5_H_4_N_4_O_3_	—	—	10.603	—
uridine	1.19	244.07	C_9_H_12_N_2_O_6_	—	−3.251	—	3.777
xanthurenic acid	0.75	205.038	C_10_H_7_NO_4_	11.37	—	—	—

Note: * Common differential metabolites in each group.

Twenty-eight differential metabolites were found in the comparison of the LNZ group and the control group (Table 3). These metabolites reflect the pharmacodynamic characteristics of 2 μg/ml LNZ in MRSA biofilms and are significantly related to pyruvate metabolism, the citrate cycle (TCA cycle), phenylalanine, tyrosine and tryptophan biosynthesis, taurine and hypotaurine metabolism and valine, leucine and isoleucine degradation (Figure 5B, Supplementary Table S4).

Twenty-five differential metabolites were found in the comparison of the 1/16RYN group and the control group (Table 3). These metabolites reflect the pharmacodynamic characteristics of 1/16 RYN treating MRSA with biofilms and are significantly related to aminoacyl-tRNA biosynthesis (Figure 5B, Supplementary Table S5).

Thirty-two differential metabolites were found in the comparison of the 2 μg/ml LNZ+1/16RYN group and the control group (Table 3). These metabolites reflect the pharmacodynamic characteristics of 2 μg/ml LNZ+16RYN treated MRSA biofilms and are significantly related to aminoacyl-tRNA biosynthesis, arginine biosynthesis, nitrogen metabolism, D-glutamine and D-glutamate metabolism and phenylalanine, tyrosine and tryptophan biosynthesis (Figure 5B, Supplementary Table S6).

### Determination of Metabolic Markers in Combination With RYN and LNZ

**FIGURE 6:**
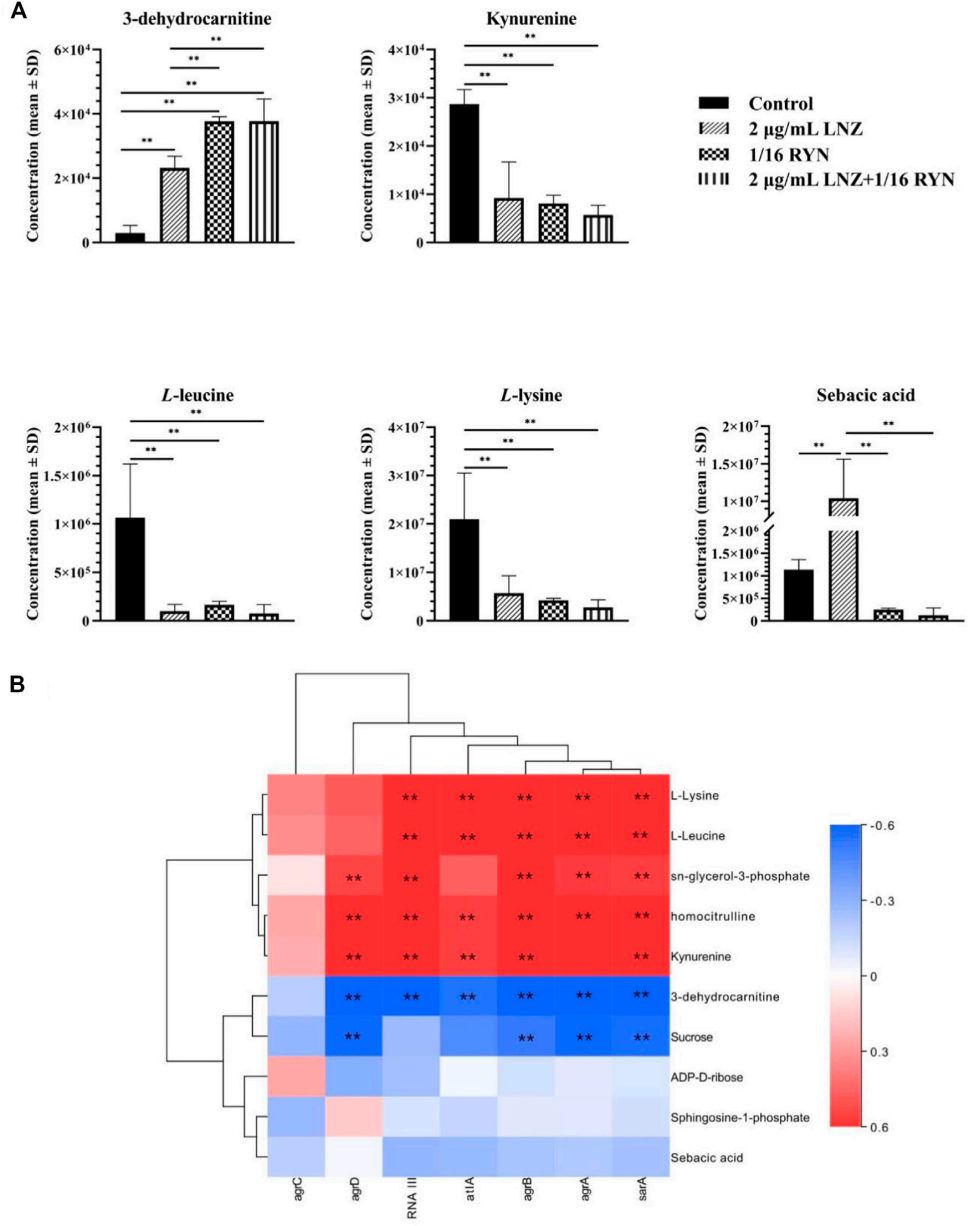
Peak intensities of pharmacodynamic biomarkers in different groups and its correlation with gene expression. **(A)** **p < 0.01. **(B)** Correlation analysis between pharmacodynamic biomarkers of RYN and LNZ and biofilm-associated genes. Significance levels are indicated as follows: **p < 0.01, red and blue indicate positive and negative correlations, respectively.

As shown in Table 3, there were 10 common metabolites in the control group, 2 μg/ml LNZ group, 1/16RYN group and 2 μg/ml LNZ+1/16RYN group. These metabolites are pharmacodynamic biomarkers of LNZ combined with RYN against MRSA. There were no metabolic pathways significantly related to these biomarkers. 3-Dehydrocarnitine decreases after biofilm formation in the control group. After intervention with 2 μg/ml LNZ and 1/16RYN alone, 3-dehydrocarnitine in cells increased. After the combined intervention of the two, 3-dehydrocarnitine increased significantly. The level of metabolites in the administration group was opposite to that in the control group, and the change trend in the combination group was consistent with that in the single group and larger than that in the single group (Figure 6A). Other metabolites similar to this change are kynurenine, *L*-leucine and *L*-lysine. Their levels increased after the biofilm matured but decreased after LNZ and RYN intervention, and the extent of the decrease increased after the combination of the two drugs. The intervention effects of LNZ and RYN on ADP-*D*-ribose, sebacic acid and sucrose were the opposite. The level of ADP-*D*-ribose decreased after biofilm formation, and LNZ intervention further promoted the reduction of ADP-*D*-ribose. The level of ADP-*D*-ribose increases after RYN intervention. The level of sebacic acid increases after the formation of biofilms, and LNZ promotes an increase in sebacic acid content. However, the level of sebacic acid decreased after RYN intervention, and increased after combined use. The level of sucrose decreases only in the LNZ group.

### Correlation Between Pharmacodynamic Biomarkers of RYN and LNZ and Biofilm-Associated Genes

As shown in Figure 6B, unsupervised clustering yielded positive correlations between L-lysine, L-leucine, kynurenine, homocitrulline, sn-glycerol-3-phosphate and seven biofilm-associated genes. 3-Dehydrocarnitine, sucrose, ADP-*D*-ribose, sphingosine-1-phosphate, and sebacic acid were negatively correlated. Correlations between *agrA*, *agrB*, and *sarA* and the ten pharmacodynamic markers were similar, and correlations between *agrC* and the ten pharmacodynamic markers were the weakest.

## Discussion

Antibiotics are currently the first-choice drugs for the clinical treatment of bacterial infections. However, for some drug-resistant bacteria, the existing antibiotics are difficult to effectively treat them. MRSA, a highly pathogenic drug-resistant bacterium, seriously threatens human health. Biofilms are the cause of MRSA resistance, enhance the pathogenicity of MRSA and make the treatment of MRSA more difficult. The lowest inhibitory concentration of the antibiotic LNZ on MRSA biofilms is 32–64 times higher than that of LNZ on planktonic MRSA biofilms (Parra-Ruiz et al., 2012). RYN, a compound preparation of TPM, is often used for the treatment of MRSA and its biofilm infection in China. Therefore, in this study we explored whether the combination of RYN and LNZ has complementary advantages and revealed its mechanisms. The results showed that the combination of drugs could synergistically inhibit the growth of MRSA and its biofilm. The mechanisms of their synergistic antibacterial action lie in affecting physiological metabolic pathways such as MRSA amino acid, fat metabolism and cell membrane synthesis by reducing the transcription of the QS system and adhesion-related genes. Eventually the combined medication reduced the dose of LNZ and inhibited the formation of biofilms.

Bacteria in biofilms and bacteria in the suspended state have large differences in growth characteristics and nutrient absorption (El-Azizi et al., 2005). The ability of MRSA to form biofilms greatly affects its survival and virulence, and gives it great resistance to host immune system clearance and clinical drug therapy. It is possible that EPS, a constituent of biofilms, may facilitate the anti-drug properties of biofilms by preventing the bulk transport of antibiotics across the biofilm in a direct drug binding manner (Donlan, 2000). In our results, the disruption of biofilms by RYN greatly assisted LNZ in exerting a bacteriostatic effect on MRSA. As a TCM compound preparation, RYN which is rich in taraxacum, emodin and polydatin has good inhibitory properties on bacteria and their biofilms (Lau and Plotkin, 2013). Emodin, an anthraquinone derivative isolated from Polygonum cuspidatum and palm wood, has inhibitory effects on the biofilm formation of *S. aureus*, *Pseudomonas aeruginosa* and *Streptococcus suis* (Yan et al., 2017). Another study found that emodin extracted from the rhizome of Polygonum cuspidatum could destroy the integrity of the MRSA cell wall and cause the loss of intracellular components, thereby significantly inhibiting the growth of MRSA strains (Cao et al., 2015). Thus, emodin may be one of the key components by which RYN inhibits MRSA and its biofilm. Existing studies have shown that the coadministration of antibiotics and nonantibiotics could reduce the resistance of bacteria and enhance the antibacterial activity of drugs (Tyski, 2003). The bacteriostatic effect of the combination of RYN and LNZ was superior to the effect of each of the two drugs alone in the *in vitro* experimental results, and the dose of the combination of RYN with LNZ was reduced compared with the dose of LNZ alone. All of these results showed that RYN acts synergistically with antibiotic drugs as a non-antibiotic drug.

**FIGURE 7:**
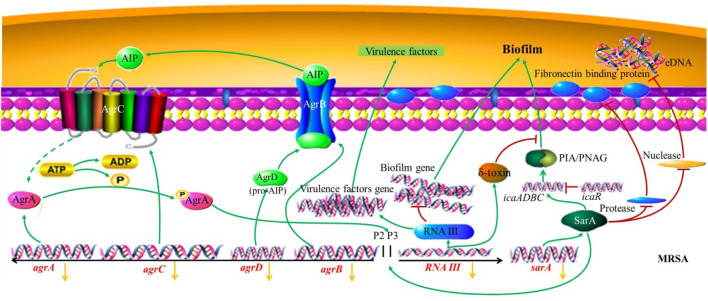
MRSA biofilm regulated by the QS system. The yellow arrow indicates that the combined action of RYN and LNZ leads to a decrease in the expression of the gene.

To further investigate the antimicrobial mechanism of RYN in combination with LNZ, we examined the QS system and adhesion genes associated with MRSA biofilm formation. Of the significantly changed genes we examined, *sarA* and *agrC* were associated with the QS system. SarA is an important transcriptional regulator in *S. aureus* that is closely related to biofilms, adhesion and haemolysis (Valle et al., 2003). Compared with planktonic bacteria, the transcription of sarA in, *S. aureus* was upregulated during the biofilm stage (Valle et al., 2003). SarA is a positive regulator of biofilm formation, in part because it inhibits the production of proteases and nucleases (Figure 7). The increase in bacterial extracellular protease can inhibit the activity of fibronectin binding protein and inhibit the adhesion of bacteria. Fibronectin binding protein is one of the surface adhesion factors of *S. aureus*, which can help bacteria adhere and colonize (Keane et al., 2007). The biofilm formation and fibronectin binding of SarA mutant strains are significantly lower than those of parent strains (Abdelhady et al., 2014). It is inferred that due to the deletion of *sarA*, the inhibition of protease by SarA disappears, the production of protease in the mutant increases, and the binding ability to fibronectin is significantly decreased, which will greatly affect the adhesion of MRSA and the production of biofilms (Abdelhady et al., 2014). In addition, the increase in nuclease production also affected the formation of biofilms due to the deletion of *sarA* (Beenken et al., 2010). Nuclease mutants could form abnormally thick biofilms, including the phenomenon of increased levels of matrix-related extracellular DNA (eDNA) (Figure 7) (Beenken et al., 2010). Bacteria release eDNA through autolysis during programmed cell death, which contributes to the formation of biofilms. The inhibition of nuclease can promote the formation of MRSA biofilms. Emodin has been reported to downregulate *sarA* and intervene in the release of eDNA (Yan et al., 2017). LNZ showed poor clearance from biofilms of *S. aureus* that were wild type for *sarA*. However, it was more sensitive to *sarA* mutant *S. aureus*, and the biofilm clearance rate was improved (Weiss et al., 2009). This suggests that SarA is an important factor in maintaining the stability of biofilms (Weiss et al., 2009). RYN caused the inhibition of SarA expression in MRSA leading to reduced MRSA biofilm production. This increases the permeability of LNZ to biofilms, thereby exerting an inhibitory effect on MRSA.

Both *agr* and *sarA* are the main regulators in biofilm formation, and the *Agr* system composed of *agr* is one of the main two-component signal transduction systems of *S. aureus*. The *Agr*-QS system showed downregulation of bacterial cell wall-associated adhesion factors, which could weaken the adhesion ability and indirectly inhibit the formation of initial biofilms (Boles and Horswill, 2008). It has been proven that inducing the expression of *agr* can lead to the separation of *S. aureus* cells from the established biofilms (Boles and Horswill, 2008). Compared with the QS system in other bacteria, *agr*, as an active QS system, hinders the development of biofilms by inhibiting the production of adhesion proteins and inducing the expression of matrix degradation enzymes (such as protease, nuclease and lipase) (Kong et al., 2006). It acts as a regulatory switch between staphylococcal planktonic cells and the biofilm state. The *Agr* system contains *agrA*, *agrB*, *agrC* and *agrD* 3 kb loci. When the bacteria reach the critical concentration, the signal molecule AIP precursor encoded by *agrD* is processed and secreted out of the cell by the AgrB transmembrane protein encoded by *agrB* (Zhang et al., 2002). AgrC, a homologous transmembrane receptor encoded by *agrC*, is the receptor of mature AIP (autoinducing peptide), and its combination can activate the autophosphorylation of transcriptional AgrA (Zhang et al., 2002). *Agr* can be induced by AIP, which is also necessary for mature biofilm dissemination (Boles and Horswill, 2008). AgrA can cooperate with SarA to participate in the activation of promoter 2 (P2) and promoter 3 (P3). P2 can regulate the *agr* system through a self-induction mechanism. P3 inhibits the formation of biofilms and turns on the expression of virulence factors by regulating the production of the *agr* effector molecule RNAⅢ (Zhang et al., 2002). As an inhibitor of protein synthesis, the concentration of LNZ used in clinical treatment has an important effect on the inhibition of *agr* activity, which can reduce the transcriptional level of *RNAⅢ* and inhibit the diseases mediated by invasive toxins caused by MRSA (Tsuji et al., 2012). This is in agreement with our experimental results. Although this inhibition of *agr* may not be conducive to the inhibition of biofilm growth, it has been reported that the effect of *sarA* on *agr* is epistatic during biofilm formation. The *agr* mutant has little effect on biofilm development *in vitro*, indicating that the biofilm is not dependent on the *agr* gene *in vitro* (Beenken et al., 2010). In addition, RNAⅢ of the P3 transcription unit of the *Agr* system can regulate virulence factors, which can help bacteria transition from the colonization stage in the early stage of infection to the stage of invasion and acquisition of host nutrients in the later stage of infection. Studies on animal infection have shown that the loss of *Agr* system function can significantly reduce the pathogenicity of *S. aureus* (Montgomery et al., 2010). This indicates that inhibition of the *Agr* system has an important role in reducing the pathogenicity of MRSA. Under the control of the QS system, the most important step to adjust bacteria from the planktonic state to the biofilm state is the adhesion between bacteria and the external environment so that bacteria can be colonized at a certain point and not easily be removed. Therefore, we examined the *atlA* gene related to MRSA adhesion. AtlA, encoded by the *atlA* gene, is the most important peptidoglycan hydrolase in staphylococci. AtlA, also known as a bifunctional autolysin precursor protein, can destroy the internal junction and cell wall structure of peptidoglycan, resulting in bacterial cell lysis and autolysis (Götz et al., 2014). In addition, the adhesion molecule-like function of AtlA is conducive to the initial adhesion between bacteria and substrate molecules, which plays an important role in the formation of biofilms and leads to the chronicity and recurrence of bacterial infection (Götz et al., 2014). Some studies have shown that the biofilm growth of *atlA* deletion mutants is inhibited (Biswas et al., 2006). In our results, the expression of *atlA* was further suppressed after the combination of the two drugs. This inhibition may lead to a decrease in the autolysis of bacteria, a decrease in the expression of AtlA protein, a decrease in the ability of bacteria to adhere to substrate molecules, and a decrease in the ability of biofilm formation. The combination of the two drugs can enhance the inhibition of the expression of *agrC*, *atlA* and *sarA*, to a certain extent, but the specific regulatory mechanism needs to be further studied.

**FIGURE 8:**
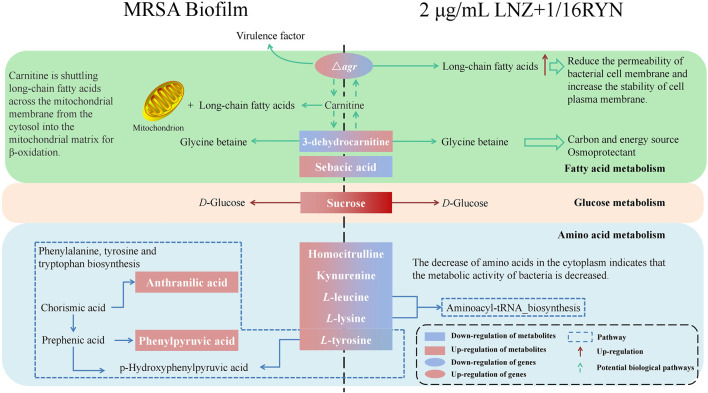
Effects of RYN combined with LNZ on MRSA metabolites. Boxes are differential metabolites and oval boxes are genes. Red boxes and red arrows indicate upregulation, and blue boxes indicate downregulation. Dashed boxes indicate pathways. Dashed arrows indicate possible potential biological pathways.

Traditional antibiotics are designed to resist planktonic bacteria and treat metabolically exuberant bacteria, but the bacteria in biofilms are different from planktonic bacteria in metabolism (Davey and O’toole, 2000). Metabolic changes contribute to a higher tolerance of bacterial biofilms to therapeutic agents (Stewart, 2002). Our data suggest that the differential metabolites of planktonic MRSA and biofilm-producing MRSA may be related to the biosynthesis of phenylalanine, tyrosine and tryptophan (Figure 5B). This is consistent with previous reports that the uptake of amino acids by biofilms is an important factor that affects biofilm physiology (Ammons et al., 2014). On this basis, we concluded that there were 10 metabolites related to the combination of RYN and LNZ in the endogenous metabolites of MRSA. Among them, the levels of homocitrulline, kynurenine, *L*-leucine and *L*-lysine decreased significantly after the combination treatment (Table 3). *S. aureus* must adapt to various carbon and nitrogen sources when invading the host. Amino acids can be used by *S. aureus* catabolism as a secondary carbon source for survival and increment (Halsey et al., 2017). In biofilms, the consumption and secretion of bacterial metabolites increased significantly. Some studies have shown that biofilm cultures of *S. aureus* selectively absorb amino acids (Ammons et al., 2014). Exposure of *S. aureus* to subtle changes in temperature, pH and osmotic pressure will lead to significant changes in the composition of amino acids in the cytoplasm of *S. aureus* (Alreshidi et al., 2016). The decrease in amino acid uptake was accompanied by a decrease in amino acids in the cytoplasm, indicating that the metabolic activity of the bacteria decreased to adapt to changes in temperature, pH and osmotic pressure (Ammons et al., 2014). According to the existing literature, by observing the catabolism of amino acids during the growth of *S. aureus*, leucine in the culture medium showed a gradual consumption trend, indicating that it may be used in protein synthesis (Alreshidi et al., 2019). The uptake of leucine by *S. aureus* was very high in both the logarithmic growth phase and stable phase, and the uptake of leucine in the stable phase was 4 times that in the logarithmic growth phase (Alreshidi et al., 2019). This is consistent with our experimental results. Under ideal conditions, leucine, lysine and serine are the most commonly used amino acids in *S. aureus* culture medium. Studies have shown that leucine deficiency could lead to a 6–8 h growth lag of *S. aureus* (Kaiser et al., 2018). The stable period is a complex period in the growth of bacteria, which is physiologically equivalent to the biofilm period (Jananee and Preeti, 2017). In the pathway analysis, it was also found that biofilm formation and drug action were closely related to the nitrogen metabolism pathway, tryptophan biosynthetic pathway and proline biosynthetic pathway (Figure 5B). It is suggested that amino acids may play an important role in the growth of MRSA biofilms. Combined with our results, it can be concluded that the combination of the two drugs may have a synergistic and additive effect on inhibiting the uptake of amino acids, which may be an important factor affecting the inhibition of MRSA biofilm growth (Figure 8).

Our study involved four different states of MRSA biofilms, including no drug intervention, 2 μg/ml LNZ intervention alone, 1/16RYN intervention alone, and 2 μg/ml LNZ+1/16RYN combined intervention. 3-dehydrocarnitine in four different states of MRSA showed that the content of 3-dehydrocarnitine decreased with the formation of biofilms without drug intervention. The content of 3-dehydrocarnitine increased after drug intervention, and the effect of the combined drug was greater than that of the single drug. 3-Dehydrocarnitine is an intermediate in the decomposition of carnitine to glycine betaine (GB) (Wargo and Hogan, 2009). Carnitine can transport long-chain fatty acids to the mitochondria of animals, so that relatively large amounts of carnitine are often present in adipose metabolic tissue (Bremer, 1983). Most bacteria can decompose carnitine into betaine (GB, proline betaine, etc.) as osmotic protective agents. Osmotic protective agents can protect bacteria against an increase in external osmotic pressure, ensure the stability of osmotic pressure inside bacterial cells and maintain cell vitality, and cell metabolism will not produce negative interference.

Carnitine can also provide carbon and nitrogen sources for bacteria (Aurich and Lorenz, 1959). In our study, we found that the content of 3-dehydrocarnitine increased significantly under the combination of LNZ and RYN. This indicated that under drug intervention, MRSA significantly produced 3-dehydrocarnitine to adapt to changes in osmotic pressure in the culture medium after administration. In addition, glucose metabolism in which sucrose is involved as an important metabolite is also one of the important aspects influenced by the association of LNZ with RYN. Internal sugars are primarily used for glycolysis, biosynthesis of various components, such as cell walls and lipoteichoic acid, and intercellular polysaccharide biosynthesis. However, high-sugar environments also tend to inhibit the growth of *staphylococci* (Luo et al., 2020).

The metabolome is downstream of the gene regulatory network and is the embodiment of biological endpoint information. Functional changes in upstream macromolecules (genes, proteins) will ultimately be reflected at the metabolic level. Changes in biofilm-associated genes will affect the levels of biofilm-associated metabolic markers. *agrA* and *agrB* genes are significantly associated with markers of metabolic pathways of amino acid metabolism and fatty acid metabolism. Current studies have shown that the level of long-chain fatty acids in the plasma membrane of the *agr* mutant strain is higher than that of the wild type (Song et al., 2020). Long-chain fatty acids help reduce cell membrane permeability and stability, which may be related to the resistance of MRSA to antibiotics (Meadows and Wargo, 2015). Carnitine transports long-chain fatty acids from the cytoplasm to the mitochondrial matrix for β-oxidation. 3-dehydrocarnitine acts as an intermediate in the breakdown of carnitine to glycine betaine, and metabolite levels may be influenced by long-chain fatty acids in the plasma membrane. It is hypothesized that the increased accumulation of long-chain fatty acids following RYN and LNZ inhibition of *agr* may be related to the increased levels of 3-dehydrocarnitine, a metabolic marker bound by RYN and LNZ. This is subject to further validation. The correlation of *sarA* with coadministered pharmacodynamic markers is similar to that of *agrA* and *agrB* (Figure 6B), but no study has yet demonstrated an association.

## Conclusion

The combination of LNZ and RYN has a synergistic inhibitory effect on MRSA and its biofilm. RYN helps LNZ break through the biofilm to come into contact with the dormant MRSA by destroying the biofilm structure and inhibiting the adhesion and aggregation of biofilms. The combined medication reduces the transcription of *agrC*, *atlA* and *sarA* and interferes with MRSA amino acid and fat metabolism to achieve the antibacterial effect of the synergistic inhibition of biofilm formation and killing of MRSA. This study lays the foundation and provides a theoretical basis for the combination of RYN and LNZ in the clinic. In further research, *in vivo* experiments will be carried out to verify the synergistic anti-MRSA and biofilm effects of RYN and LNZ to solve the problem of the clinical treatment of MRSA and its biofilm infections.

## Data Availability

The original contributions presented in the study are included in the article/Supplementary Materials, further inquiries can be directed to the corresponding authors.

## Abbreviations

CLSM, Confocal laser scanning microscopy; eDNA, extracellular DNA; EPS,Extracellular polymeric substances; FC, Fold change; FICI, Fractional inhibition concentration index; GB, glycine betaine; LB, Luria-Bertani; LNZ, linezolid; MIC, Minimum inhibitory concentrations; MRSA, Methicillin-resistant *Staphylococcus aureus*; PCA, Principal component analysis; PMS, Phenazine methosulfate; QS, Quorum sensing; RT–PCR, Real-time fluorescence polymerase chain reaction; RYN, Reyanning mixture; SEM, Scanning electron microscope; TPM, Traditional plant medicine; TTC, 2,3,5-triphenyltetrazolium chloride; XTT, 2,3-bis-(2-methoxy-4-nitro-5-sulfophenyl)-2H-tetrazolium-5-carboxanilide.
