# Supplementary material for: The Inhibition Effect of Linezolid With Reyanning Mixture on MRSA and its Biofilm is More Significant than That of Linezolid Alone
Source: Front Pharmacol. 2022 Jan 3;12:766309. doi: 10.3389/fphar.2021.766309 (PMC8762264; doi:10.3389/fphar.2021.766309)
Supplement: Supplementary file 1 [file Table1.DOCX]

**Table 1. Primers used in this study**

| **Primer name** | **Primer sequence (5'to3')** | **Product size（bp）** |
| --- | --- | --- |
| *agrA*-F | TGTTATCAATGGTCACTTATGCTG | 323 |
| *agrA*-R | GTTTGCTTCAGTGATTCGTTTATT |  |
| *agrB*-F | AGTACGTTTAGGGATGCAGGTC | 176 |
| *agrB*-R | CCACATAACACCAAAATGAAGAAG |  |
| *agrC*-F | CTTGATAACGCAATAGAGGCA | 179 |
| *agrC*-R | CCTAAACCACGACCTTCACC |  |
| *agrD*-F | TCATTTTTTGATTTTATAACTGGTG | 101 |
| *agrD*-R | TCTTTAGGTATTTCAACTTCGTCC |  |
| *atlA*-F | ACGTGTACCAGGTAAGTGGACAGA | 191 |
| *atlA*-R | AATGCTGGATCTTGAGCTAAACG |  |
| *RNA III*-F | TAAACATCCCAACTTGCCAGA | 205 |
| *RNA III*-R | ATCCAAATACAATGCCCCAAT |  |
| *sarA*-F | ATGGGGAACATGATCCTTTG | 294 |
| *sarA*-R | TAGCCGCATAACGAGCAGTA |  |
| 16s rRNA-F | TTCTGGTCTGTAACTGACGCTG | 299 |
| 16s rRNA-R | CGAAGGGGAAGGCTCTATCT |  |

**Table 2. The MIC, FICI and action mode of LNZ and RYN on MRSA**

| Strain | LNZ (μg/mL) | | RYN | | FICI | Outcome |
| --- | --- | --- | --- | --- | --- | --- |
|  | Alone | Combination | Alone | Combination |  |  |
| MRSA | 4 | 2 | 1/2 | 1/32 | 0.625 | Addition |

**Table 3. Different metabolites in each group**

| **Compound** | **R.T (min)** | **Exact mass** | **Formula** | **Fold change** | | | |
| --- | --- | --- | --- | --- | --- | --- | --- |
|  |  |  |  | **Control** | **2μg/mL LNZ** | **1/16RYN** | **2μg/mL LNZ+1/16RYN** |
| 1,3,7-trimethyluric acid | 5.96 | 210.075 | C_8_H_10_N_4_O_3_ | 5.925 | - | - | - |
| 1-O-caffeoylglucose | 1.98 | 342.095 | C_15_H_18_O_9_ | - | - | - | -5.034 |
| 2-methylbutanoyl-coenzyme A | 5.36 | 851.173 | C_26_H_44_N_7_O_17_P_3_S | 7.369 | 5.391 | - | -4.962 |
| 2-succinylbenzoyl-coenzyme A | 1.09 | 971.158 | C_32_H_44_N_7_O_20_P_3_S | -7.372 | 5.004 | - | - |
| 3-dehydrocarnitine* | 4.74 | 160.097 | C_7_H_13_NO_3_ | -9.932 | 5.758 | 5.307 | 12.805 |
| acetyl phosphate | 0.90 | 139.988 | C_2_H_5_O_5_P | 10.32 | -4.781 | - | - |
| acetyl-coenzyme A | 3.81 | 809.126 | C_23_H_38_N_7_O_17_P_3_S | - | 3.86 | - | - |
| ADP-D-ribose* | 0.67 | 559.072 | C_15_H_23_N_5_O_14_P_2_ | -5.387 | -11.314 | 4.462 | 4.324 |
| anthranilic acid | 0.79 | 137.048 | C_7_H_7_NO_2_ | 6.859 | - | - | - |
| bis (3',5')-cyclic diguanylic acid | 1.26 | 690.095 | C_20_H_24_N_10_O_14_P_2_ | - | - | 3.652 | - |
| citrulline | 6.36 | 175.096 | C_6_H_13_N_3_O_3_ | - | - | -5.688 | -5.667 |
| cyclic AMP | 1.75 | 329.053 | C_10_H_12_N_5_O_6_P | 4.493 | - | - | -4.947 |
| cytidine | 1.10 | 243.086 | C_9_H_13_N_3_O_5_ | - | 6.889 | - | - |
| cytosine | 0.84 | 111.043 | C_4_H_5_N_3_O | -6.288 | - | 3.139 | - |
| *D*-alanine | 1.10 | 89.048 | C_3_H_7_NO_2_ | - | -4.322 | - | - |
| D-butyrine | 0.73 | 103.063 | C_4_H_9_NO_2_ | -4.792 | - | - | - |
| dCMP | 0.71 | 307.057 | C_9_H_14_N_3_O_7_P | 6.882 | - | - | - |
| dephospho-coenzyme A | 3.64 | 687.149 | C_21_H_35_N_7_O_13_P_2_S | - | 4.549 | 12.092 | - |
| *D*-erythro-dihydrosphingosine | 10.32 | 301.298 | C_18_H_39_NO_2_ | - | 6.844 | - | - |
| D-lactic acid | 0.72 | 90.032 | C_3_H_6_O_3_ | 4.197 | -4.223 | - | - |
| dTMP | 0.89 | 322.057 | C_10_H_15_N_2_O_8_P | - | - | - | 3.784 |
| gamma-glutamyl phosphate | 6.79 | 227.02 | C_5_H_10_NO_7_P | - | -3.496 | - | - |
| glycerol | 9.70 | 92.047 | C_3_H_8_O_3_ | - | - | - | 3.256 |
| glycocholic acid | 8.86 | 465.309 | C_26_H_43_NO_6_ | - | - | 4.7 | - |
| histamine | 0.76 | 111.08 | C_5_H_9_N_3_ | -3.219 | - | - | - |
| homocitrulline* | 6.99 | 189.111 | C_7_H_15_N_3_O_3_ | 3.616 | -3.132 | -3.955 | -3.869 |
| inosine | 7.26 | 268.081 | C_10_H_12_N_4_O_5_ | - | -5.497 | -4.391 | - |
| kynurenine* | 2.08 | 208.085 | C_10_H_12_N_2_O_3_ | 3.41 | -3.112 | -3.555 | -5.055 |
| L-carnitine | 0.77 | 161.105 | C_7_H_15_NO_3_ | -4.065 | - | - | - |
| *L*-cystathionine | 0.70 | 222.067 | C_7_H_14_N_2_O_4_S | - | - | - | 4.573 |
| L-glutamic acid | 0.73 | 147.053 | C_5_H_9_NO_4_ | -7.229 | - | - | 6.018 |
| *L*-glutamine | 2.27 | 146.069 | C_5_H_10_N_2_O_3_ | - | -3.267 | - | -6.258 |
| *L*-histidine | 0.81 | 155.07 | C_6_H_9_N_3_O_2_ | - | - | -5.45 | -5.502 |
| *L*-leucine* | 1.09 | 131.095 | C_6_H_13_NO_2_ | 0.377 | -10.794 | -6.502 | -14.485 |
| *L*-lysine* | 0.77 | 146.106 | C_6_H_14_N_2_O_2_ | 0.531 | -3.684 | -4.973 | -7.645 |
| *L*-malic acid | 0.83 | 134.022 | C_4_H_6_O_5_ | - | 13.672 | - | - |
| *L*-phenylalanine | 3.74 | 165.079 | C_9_H_11_NO_2_ | - | - | - | -3.613 |
| *L*-proline | 0.78 | 115.063 | C_5_H_9_NO_2_ | - | - | 4.285 | 4.521 |
| L-sorbose | 0.76 | 180.063 | C_7_H_14_O_5_ | 3.127 | - | -6.449 | - |
| *L*-tryptophan | 8.89 | 204.09 | C_11_H_12_N_2_O_2_ | - | -3.136 | - | -4.905 |
| L-tyrosine | 1.13 | 181.074 | C_9_H_11_NO_3_ | 5.083 | -5.689 | - | -9.918 |
| L-urobilin | 4.70 | 594.342 | C_33_H_46_N_4_O_6_ | 3.156 | - | - | - |
| N-acetyl-D-phenylalanine | 5.54 | 207.09 | C_11_H_13_NO_3_ | 4.615 | - | -5.425 | - |
| phenylpyruvic acid | 1.14 | 164.047 | C_9_H_8_O_3_ | 7.009 | 3.852 | 5.721 | - |
| phosphorylcholine | 3.71 | 169.05 | C_4_H_12_NO_4_P | - | - | - | -4.34 |
| pyrrolidonecarboxylic acid | 0.85 | 129.043 | C_5_H_7_NO_3_ | -7.133 | - | - | 5.791 |
| ribose | 0.73 | 150.053 | C_5_H_10_O_5_ | - | - | 4.481 | 4.761 |
| S-adenosylmethionine | 7.24 | 398.137 | C_15_H_22_N_6_O_5_S | - | - | 8.838 | 7.276 |
| sebacic acid* | 0.90 | 202.121 | C_10_H_18_O_4_ | 3.932 | 9.14 | -4.511 | -9.166 |
| sn-glycerol-3-phosphate* | 0.70 | 172.014 | C_3_H_9_O_6_P | 4.181 | -3.816 | -10.373 | -9.086 |
| sphingosine-1-phosphate* | 1.95 | 379.249 | C_18_H_38_NO_5_P | 2.548 | -10.714 | -10.876 | -8.764 |
| succinic acid | 1.14 | 118.027 | C_4_H_6_O_4_ | - | 7.623 | - | 13.879 |
| sucrose* | 1.07 | 342.116 | C_12_H_22_O_11_ | 6.765 | -3.593 | 3.954 | 3.546 |
| taurolithocholate-3-sulfate | 4.29 | 563.259 | C_26_H_45_NO_8_S_2_ | - | - | - | -7.35 |
| trans-oct-2-enoyl-coenzyme A | 1.10 | 891.204 | C_29_H_48_N_7_O_17_P_3_S | -6.914 | - | - | - |
| tyrosine methyl ester | 10.24 | 195.09 | C_10_H_13_NO_3_ | - | - | - | 4.83 |
| uric acid | 0.98 | 168.028 | C_5_H_4_N_4_O_3_ | - | - | 10.603 | - |
| uridine | 1.19 | 244.07 | C_9_H_12_N_2_O_6_ | - | -3.251 | - | 3.777 |
| xanthurenic acid | 0.75 | 205.038 | C_10_H_7_NO_4_ | 11.37 | - | - | - |

Note: * Common differential metabolites in each group.
