# Supplementary material for: The Inhibition Effect of Linezolid With Reyanning Mixture on MRSA and its Biofilm is More Significant than That of Linezolid Alone
Source: Front Pharmacol. 2022 Jan 3;12:766309. doi: 10.3389/fphar.2021.766309 (PMC8762264; doi:10.3389/fphar.2021.766309)
Supplement: Supplementary file 2 [file Table2.DOCX]

1. **Quality identification of the finished product of Reyanning mixture (RYN)**

According to the regulations of the Chinese Pharmacopoeia, we used fingerprints for quality control and identification of RYN.

**
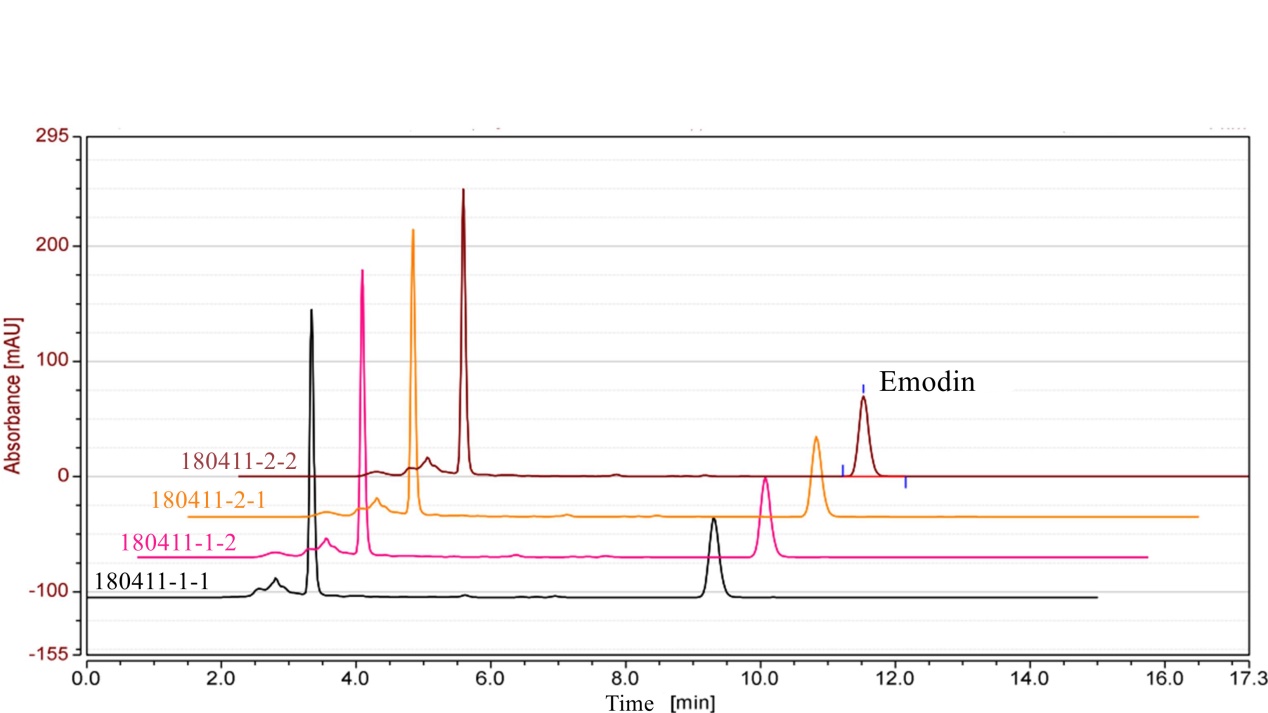
**

**Figure 1. Chromatograms of emodin quality control.**

**Table 1. Chromatographic results for emodin quality control**

| **Name** | **Retention time (min)** | **Peak area (mAU*min)** | **Sample concentration**  **(mg/mL)** | **Asymmetry factor** | **Theoretical plate number** |
| --- | --- | --- | --- | --- | --- |
| 180411-1-1 | 9.308 | 12.485 | 0.949 | 1.12 | 17712 |
| 180411-1-2 | 9.320 | 12.469 | 0.9477 | 1.13 | 17797 |
| 180411-2-1 | 9.328 | 12.468 | 0.9477 | 1.13 | 17811 |
| 180411-2-2 | 9.278 | 12.459 | 0.9496 | 1.12 | 17734 |
| Sum | 37.235 | 49.881 | 3.791 | 4.509 | 71054 |
| Average value | 9.309 | 12.470 | 0.948 | 1.127 | 17763.5 |
| Relative standard deviation | 0.235% | 0.089% | 0.089% | 0.458% | 0.270% |

**
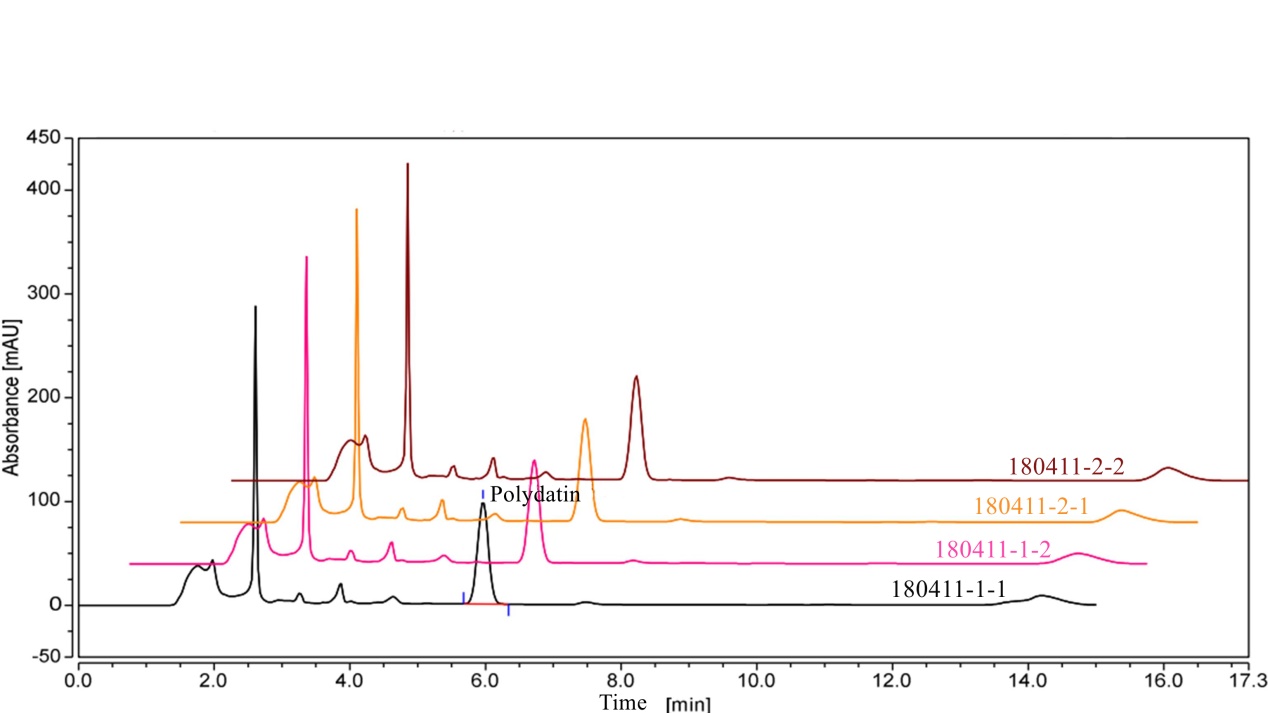
**

**Figure 2. Chromatograms of polydatin quality control.**

**Table 2. Chromatographic results for polydatin quality control**

| **Name** | **Retention time (min)** | **Peak area (mAU*min)** | **Sample concentration**  **(mg/mL)** | **Asymmetry factor** | **Theoretical plate number** |
| --- | --- | --- | --- | --- | --- |
| 180411-1-1 | 5.965 | 18.554 | 2.4414 | 1.00 | 6300 |
| 180411-1-2 | 5.968 | 18.567 | 2.4431 | 1.00 | 6413 |
| 180411-2-1 | 5.970 | 18.493 | 2.4333 | 1.00 | 6410 |
| 180411-2-2 | 5.972 | 18.568 | 2.4432 | 1.00 | 6516 |
| Sum | 23.874 | 74.181 | 9.761 | 3.999 | 25639 |
| Average value | 5.969 | 18.545 | 2.44 | 1.000 | 6409.75 |
| Relative standard deviation | 0.048% | 0.192% | 0.192% | 0.370% | 1.376% |

1. **The minimum inhibitory concentration (MIC) value of Linezolid (LNZ) and RYN to MRSA and the result of chessboard method**

**
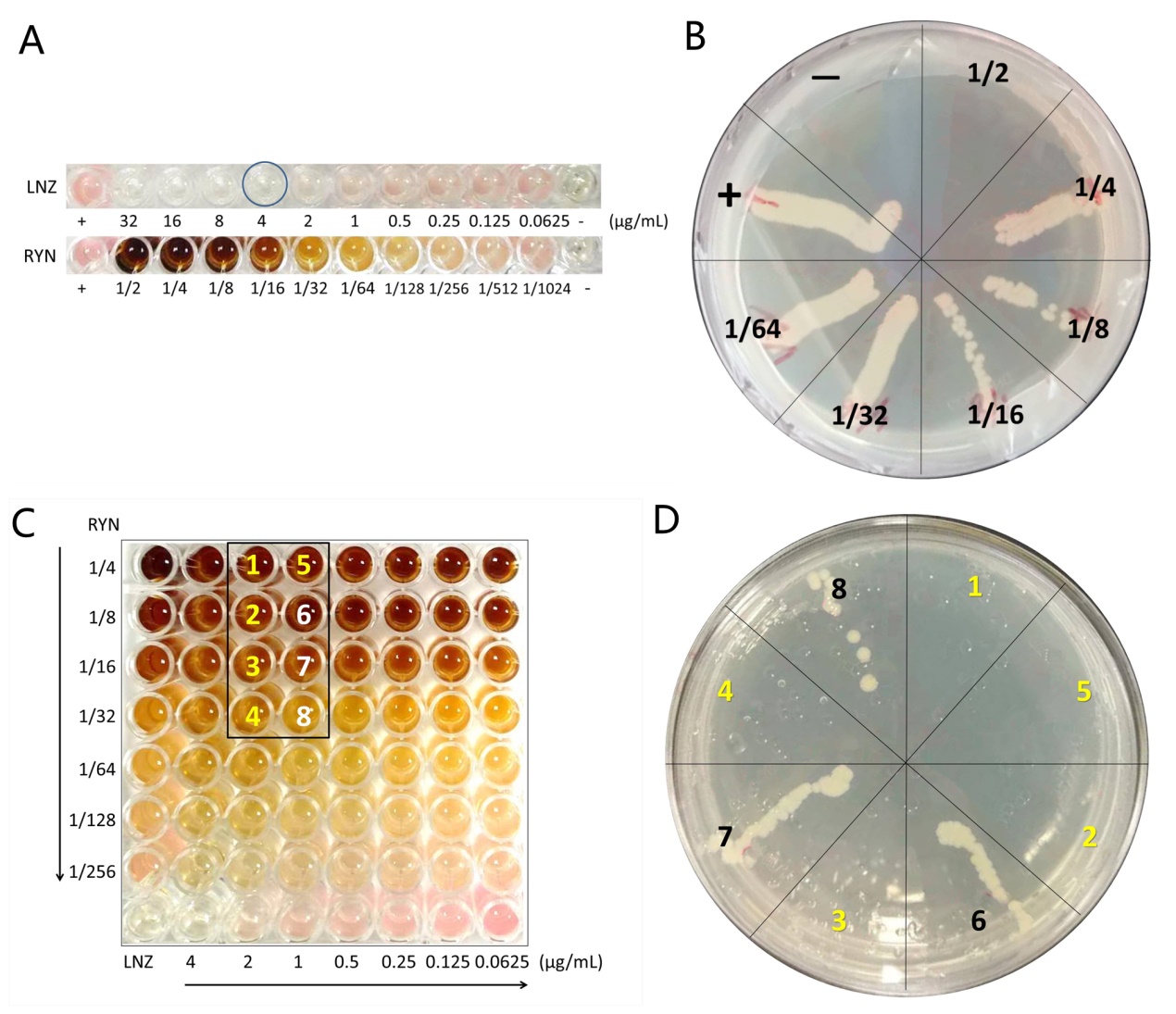
**

**Figure 3. The MICs and checkerboard results of LNZ and RYN.** A. The MICs of LNZ and RYN. B. The result of streaking on the LB agar plate of the RYN test well in A. C. The checkerboard results of LNZ and RYN. D. The results of streaking on the LB agar plate of the RYN test well in C.

1. **RNA extraction and amplification results**

The electrophoresis results show that the bands of each group are intact and there is no trailing phenomenon, indicating that the extracted RNA have no degradation and can be used for later experiments (Figure 4).

**
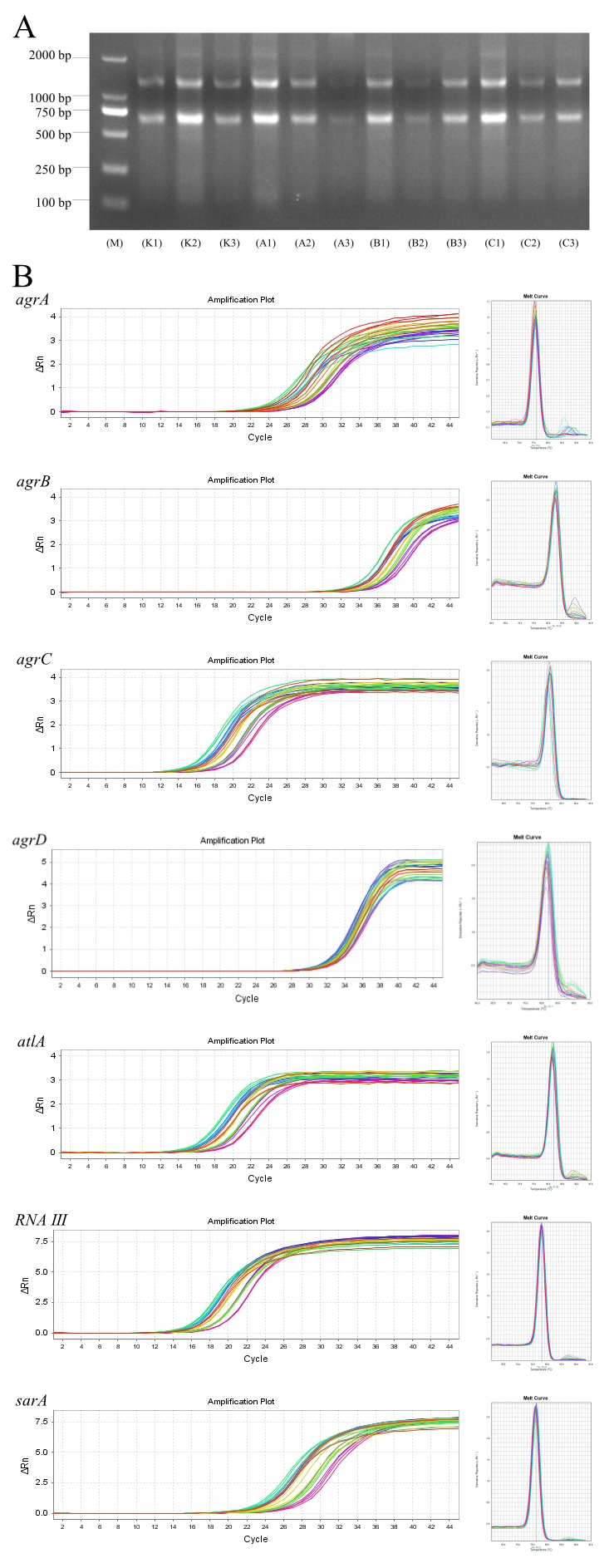
**

**Figure 4. Denaturing agarose gel electrophoresis and amplification and melt curve of RNA.** A. RNA agarose gel electrophoresis results. (M): DNA Maker, (K1)-(K3): Control, (A1)-(A3): 2μg/mL LNZ, (B1)-(B3): 1/16 RYN, (C1)-(C3): 2μg/mL LNZ+1/16RYN. B. RNA real-time amplification curve and the melt curve of amplified products.

**4. Analysis of pathways related to MRSA differential metabolites in each group**

**Table 3. Metabolic Pathways relevant to twenty-seven biofilm biomarkers**

| **Pathway Name** | **Match Status** | ***P*** | **-log(*P*)** | **FDR** |
| --- | --- | --- | --- | --- |
| Phenylalanine, tyrosine and tryptophan biosynthesis | 3/22 | 0.029658 | 3.518 | 1.0 |
| Taurine and hypotaurine metabolism | 2/9 | 0.030725 | 3.4827 | 1.0 |

**Table 4. Metabolic Pathways relevant to pharmaceutical markers of 2μg/mL LNZ intervention on MRSA biofilm**

| **Pathway Name** | **Match Status** | ***P*** | **-log(*P*)** | **FDR** |
| --- | --- | --- | --- | --- |
| Pyruvate metabolism | 4/23 | 0.0055165 | 5.2 | 0.41374 |
| Citrate cycle (TCA cycle) | 3/20 | 0.025471 | 3.6702 | 0.62036 |
| Phenylalanine, tyrosine and tryptophan biosynthesis | 3/22 | 0.032918 | 3.4137 | 0.62036 |
| Taurine and hypotaurine metabolism | 2/9 | 0.033086 | 3.4086 | 0.62036 |
| Valine, leucine and isoleucine degradation | 3/25 | 0.046012 | 3.0788 | 0.69019 |

**Table 5. Metabolic Pathways relevant to pharmaceutical markers of 1/16RYN intervention on MRSA biofilm**

| **Pathway Name** | **Match Status** | ***P*** | **-log(*P*)** | **FDR** |
| --- | --- | --- | --- | --- |
| Aminoacyl-tRNA biosynthesis | 4/45 | 0.027764 | 3.584 | 1.0 |

**Table 6. Metabolic Pathways relevant to pharmaceutical markers of 2μg/mL LNZ+1/16 RYN intervention on MRSA biofilm**

| **Pathway Name** | **Match Status** | ***P*** | **-log(*P*)** | **FDR** |
| --- | --- | --- | --- | --- |
| Aminoacyl-tRNA biosynthesis | 8/45 | 1.4292E-4 | 8.8532 | 0.010719 |
| Arginine biosynthesis | 3/16 | 0.020299 | 3.8972 | 0.49598 |
| Nitrogen metabolism | 2/7 | 0.026453 | 3.6324 | 0.49598 |
| *D*-Glutamine and *D*-glutamate metabolism | 2/7 | 0.026453 | 3.6324 | 0.49598 |
| Phenylalanine, tyrosine and tryptophan biosynthesis | 3/22 | 0.047778 | 3.0412 | 0.71667 |
